# Machine learning metrology of cell confinement in melt electrowritten three-dimensional biomaterial substrates

**DOI:** 10.1038/s41378-019-0055-4

**Published:** 2019-03-25

**Authors:** Filippos Tourlomousis, Chao Jia, Thrasyvoulos Karydis, Andreas Mershin, Hongjun Wang, Dilhan M. Kalyon, Robert C. Chang

**Affiliations:** 1 The Center for Bits and Atoms, Massachusetts Institute of Technology, Cambridge, MA USA; 2 Biomedical Engineering Department, Stevens Institute of Technology, Hoboken, NJ USA; 3 Chemical Engineering and Materials Science Department, Stevens Institute of Technology, Hoboken, NJ USA; 4 Mechanical Engineering Department, Stevens Institute of Technology, Hoboken, NJ USA

**Keywords:** Materials science, Engineering

## Abstract

Tuning cell shape by altering the biophysical properties of biomaterial substrates on which cells operate would provide a potential shape-driven pathway to control cell phenotype. However, there is an unexplored dimensional scale window of three-dimensional (3D) substrates with precisely tunable porous microarchitectures and geometrical feature sizes at the cell’s operating length scales (10–100 μm). This paper demonstrates the fabrication of such high-fidelity fibrous substrates using a melt electrowriting (MEW) technique. This advanced manufacturing approach is biologically qualified with a metrology framework that models and classifies cell confinement states under various substrate dimensionalities and architectures. Using fibroblasts as a model cell system, the mechanosensing response of adherent cells is investigated as a function of variable substrate dimensionality (2D vs. 3D) and porous microarchitecture (randomly oriented, “non-woven” vs. precision-stacked, “woven”). Single-cell confinement states are modeled using confocal fluorescence microscopy in conjunction with an automated single-cell bioimage data analysis workflow that extracts quantitative metrics of the whole cell and sub-cellular focal adhesion protein features measured. The extracted multidimensional dataset is employed to train a machine learning algorithm to classify cell shape phenotypes. The results show that cells assume distinct confinement states that are enforced by the prescribed substrate dimensionalities and porous microarchitectures with the woven MEW substrates promoting the highest cell shape homogeneity compared to non-woven fibrous substrates. The technology platform established here constitutes a significant step towards the development of integrated additive manufacturing—metrology platforms for a wide range of applications including fundamental mechanobiology studies and 3D bioprinting of tissue constructs to yield specific biological designs qualified at the single-cell level.

## Cell morphology: Fabricating 3-D biomaterial substrates using melt electrowriting

Melt electrowriting (MEW) is an advanced manufacturing process for fabricating biomaterial substrates, and in conjunction with a machine-learning algorithm to classify cell shapes, enables a cell-based model with high cell shape homogeneity. Adjusting cell shape by altering the biophysical properties of the biomaterial substrates on which cells operate is a potential way of programming cellular morphology and function. Using fibroblasts (connective tissue cells) as a model, a team headed by Robert C. Chang at the Stevens Institute of Technology in Hoboken, US, found that precision-stacked (“woven”) MEW substrates yielded high cell shape homogeneity. The team, including lead author Filippos Tourlomousis, believes that its technology platform represents a major step toward a wide range of applications, including fundamental mechanobiology studies and 3-D printing of tissue constructs to meet specific biological designs at the single-cell level.

## Introduction

Cells sense physical aspects of their local microenvironment and respond accordingly by acquiring specific phenotypes over time that are tightly related to their function, indicating that an intimate link exists between cell shape and function^1–3^. The existence of an “inside-out” mechanism has been demonstrated, whereby global cell shape distortion produces increased tension in the cell’s internal scaffolding that, in turn, feeds back to drive local changes in the assembly of shape-bearing adhesion proteins, i.e., focal adhesions (FAs)^4^. FAs function not only as anchors that structurally link cells to the material matrix, but also as signal transduction elements that relay signals from the local microenvironment into the cytoplasm^5,6^.

The principle of controlling cell function through cell shape manipulation has led to the development of engineered culture models made from natural or synthetic polymers^7–11^. In general, hydrogel-based systems with tunable stiffness parameter are considered the gold standard for three-dimensional (3D) cell culture^12,13^. Biological gels composed of in vivo proteins have indeed yielded significant dimensional and architecture-dependent differences with concomitant alterations in cellular responses^14–18^. However, the non-reproducible nature of these systems due to the local substrate remodeling associated with cell migration renders them non-ideal as culture models for cellular mechanosensing studies^19^. One possible method involves the fabrication of functionalized non-woven gel electrospun fiber meshes followed by in situ cross-linking for stiffness control^20^. However, the chaotic nature of the electrospinning process dynamics, which is responsible for uniaxial fiber stretching and the formation of high surface to volume ratio meshes, does not offer precision control over the fibrous architecture. Thus, there is a need for 3D culture models with well-defined cellular-relevant geometrical feature sizes that can decouple stiffness from the architecture of the substrate as well as provide tight control over the porous architecture at the single cell level.

To address this need, the method of melt electrowriting (MEW), a structured fibrous substrate fabrication process, inspired by the direct writing of solution electrospun fibers^21,22^ is introduced to provide the precision-stacking of highly stiff microscale fibers (made from polycaprolactone (PCL))^23–25^. The biological relevance of the fabricated substrates is demonstrated by culturing human adherent cells on stiff substrates with varying dimensionality and architecture. The resultant cell morphologies are compared for different substrate geometries. The ability of MEW to induce natural cell morphologies, owing to their confinement and suspension states within the local 3D porous microenvironment of the fabricated substrates, is demonstrated. Furthermore, a machine learning-based metrology framework is developed and applied to probe the effects of substrate architectures on cell shape and FA protein distributions. This framework enables metrics to be defined based on cell and sub-cellular FA protein features as measured using confocal fluorescence microscopy in conjunction with an automated single-cell bioimage data analysis workflow. Single-cell confinement states are implemented as a multi-dimensional metric composed of the previously extracted metrics to train and design a classifier. This strategy allows quantitative inference that the observed confinement states directly map to each substrate dimensionality and architecture.

## Results

### Fabrication of polymeric substrates with fibrous architecture

**Fig. 1:**
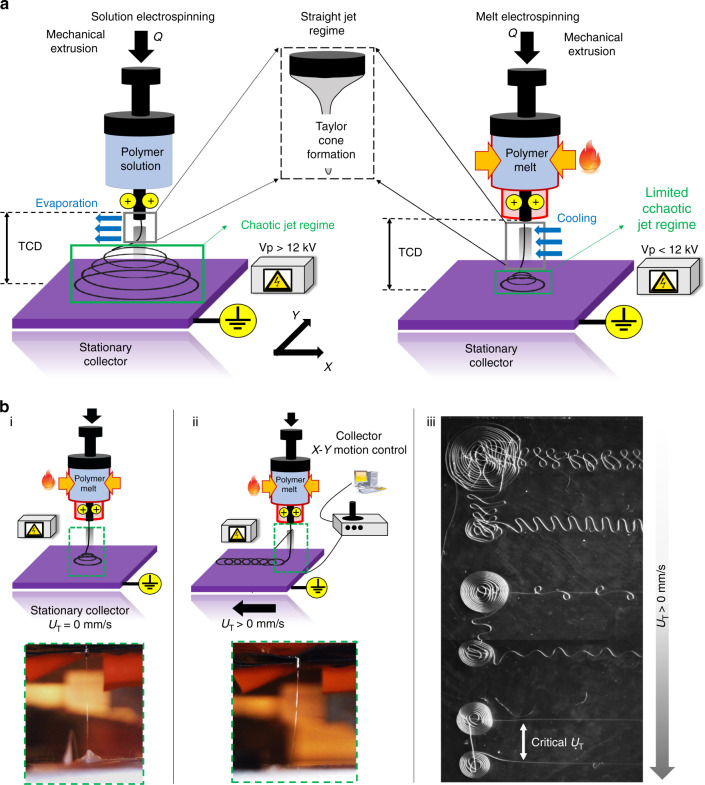
Electrohydrodynamics (EHD)-based fabrication methods employed in this study. **a** Solution electrospinning (SES) vs. melt electrospinning (MES). The main differentiating feature between the two processes is the extent of the jet instabilities that arise from the electrostatic forces acting at the polymer jet-air interface. For MES, the chaotic jet regime is limited close to the grounded collector plate due to the high viscosity and dielectric properties of the pure polymer melt. **b** Direct melt electrowriting (MEW) and its operating principle. (i) 3D conical fiber structures are obtained by the layered deposition of fibers in circular patterns due to jet instabilities close to the stationary collector plate. (ii) The jet instabilities can be eliminated by moving the grounded collector plate at prescribed translational stage speeds. (iii) Micrograph depicting various fiber topographies that are obtained by tuning the translational stage speed (*U*_T_ [SI: mm/s)]. Coiling fiber structures are obtained for the lowest *U*_T_ setting. Coiling frequency of these fiber structures can be gradually eliminated by gradually increasing *U*_T_ to achieve aligned fibers at the critical *U*_T_ setting

The first part of the study is aimed at engineering substrates with fiber-based structural features. Substrates of variable dimensionality and architecture are fabricated on flat glass coverslips using solution electrospinning (SES) and MEW. Concept schematics of both fabrication processes are depicted in Fig. 1.

The SES technique has been widely used in tissue engineering applications^26–32^ and is also used in this study to generate PCL fibrous meshes with random fiber topography (“non-woven”). The operating principle underlying the widely used conventional electrospinning process is extruding and electrostatically drawing a polymer solution or melt between a positively charged needle tip and a grounded stationary or mobile collector. The independent process parameters are the volumetric flow rate (*Q*), voltage potential (Vp), tip to collector distance (TCD), and temperature at the needle tip (*T*) (Fig. 1a). Generally, trial and error methods are utilized for the determination of suitable operating conditions which are systematically varied until the electrostatic stresses acting at the polymer solution–air interface can overcome the surface tension and the elasticity of the polymer, leading to the formation of a stable Taylor cone and the generation of fibers with targeted diameters (Fig. 1a, b).

**Fig. 2:**
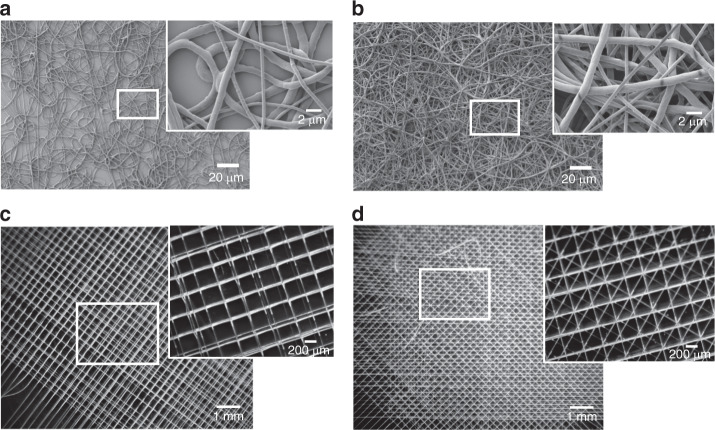
Fibrous mesh morphologies employed in this study. **a** 2D non-woven fibrous mesh fabricated with solution electrospinning (SES) and a prescribed spinning time set of 1 min. The sample is designated as SES-1 min. **b** 2D non-woven fibrous mesh fabricated with SES and a prescribed spinning time set of 3 min. The sample is designated as SES-3 min. **c** 3D woven fibrous mesh with “0–90°” pore microarchitecture fabricated with direct melt electrowriting (MEW). The sample is designated MEW|0–90°. **d** 3D woven fibrous mesh with “0–45°” pore microarchitecture. The sample is designated as MEW|0–45°

The stability of the Taylor cone combined with the chaotic motion of the fiber upon release from the Taylor cone (Fig. 1a) allows the fabrication of non-woven fibrous meshes with relatively uniform fiber diameters and with typically high surface to volume ratios. The typical conditions used for the generation of solution electrospun meshes are as follows: *Q* = 10 μL/min, Vp = 15 kV, and TCD = 15 cm (see Materials and methods for details). For the current study, the implemented strategy and process parameter settings have previously been described^33,34^. Initially, the applied voltage potential (Vp—[kV]) is tuned in combination with the needle TCD (*d*—[cm]) in order to achieve jet formation for the prescribed PCL concentration (12% in hexafluoroisopropanol (HFIP)). After obtaining a Taylor cone jet, the volumetric flow (*Q*—[mL/h]) rate is tuned in order to stabilize the jet and generate fibers, eliminating the well-known “beading phenomenon” (irregularities across the fiber length characterized by non-stretched material). While performing this tuning procedure assures a stable cone-jet for a short length, there is a secondary chaotic process regime of much larger length. During this regime, the jet experiences high-frequency whipping instabilities undergoing bending and excessive stretching^35^. To overcome this, studies have demonstrated that using a more conductive solvent can lead to more uniform charge distribution along the spinning jet and thus more uniform stretching of the fiber jet^36^. Tuning the electrospinning time provides control over the collected fiber density. It is observed that when an electrospinning time of 1 min is prescribed, thin substrates (hereafter designated as SES-1 min) exhibiting discontinuous patches without any fiber coverage are obtained (Fig. 2a). On the other hand, when the spinning time is increased to 3 min, substrates (hereafter designated as SES-3 min) exhibit uniform fiber coverage (Fig. 2b). Thus, with these two experimental runs, electrospun meshes with random pore microarchitectures possessing different pore size distributions are fabricated.

3D PCL fibrous meshes with precision-stacked fiber topographies (“woven”) are fabricated using an in-house developed MEW process design. MEW is a two-stage hybrid materials processing technique that integrates the melt electrospinning process with melt extrusion-based additive manufacturing (3-D printing) methods^25^. During melt electrospinning, the instabilities arising from the electrostatic nature of the process are limited to a much smaller regime (Fig. 1b) compared to that observed during SES (Fig. 1a). This arises due to the high viscosity and low electrical conductivity of the polymer melt. We have shown earlier that the printing of fibrous mesh structures with precise geometries can be achieved provided that the process parameters are properly optimized^37^. MEW provides the capability of generating precision-stacked fibrous meshes with fiber diameters as low as 10 μm, which is not possible with the conventional 3-D printing methods.

In the present study, a similar parameter tuning procedure is used to determine the optimum printability conditions. The objective is to achieve straight charged jets (whipping constrained to the vicinity of the collecting plate) during melt electrospinning so that the subsequently applied second component of the hybrid process, i.e., the 3-D printing, can generate precisely-stacked fibrous structures. In this procedure, it is important to balance the downstream pulling on the fiber with the upstream resistive forces. This process parameterization, in tandem with the tuning of the translational stage speed at its critical value, yields a steady equilibrium printing state characterized by precise fiber placement of aligned fibers. At the optimum MEW settings (Vp = 11 kV, *Q* = 15 μL/h, *U*_T_ = 60 mm/s, *T* = 78 °C), precisely stacked fibrous meshes with well-defined pore architectures can be printed. The specific toolpaths followed by the translational x–y stage of the printing system are patterned to generate two types of precisely-stacked meshes designated as either MEW|0–90° (Fig. 2c) and MEW|0–45° (Fig. 2d). The readily evident precise nature of the stacking of the fibers should be noted.

**Fig. 3:**
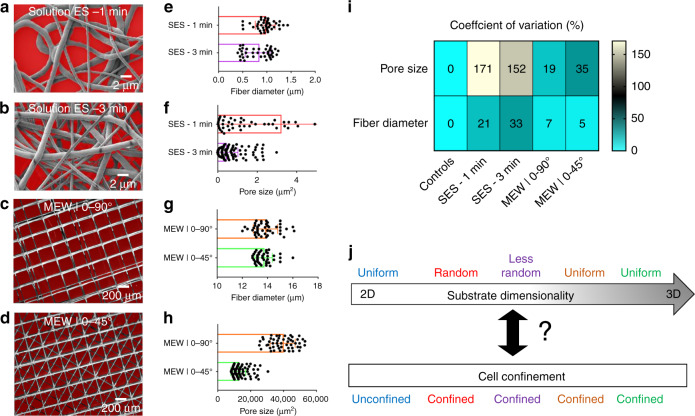
Dimensional metrology of fibrous meshes. **a**, **b** SEM micrographs of 2D nonwoven fibrous meshes overlaid with segmented effective pore size areas (red color). **c**, **d** SEM micrographs of the 3D woven fibrous meshes with different architectures, overlaid with segmented effective pore size areas (red color). **e**–**h** Bar graphs depicting the mean fiber diameter ([μm], SI) and the mean effective pore size area along with standard deviation based on the image-based segmentation results for each 2D non-woven fiber mesh (**e**, **f**) and 3D woven fiber mesh (**g**, **h**). Mean and standard deviation values are calculated based on random individual fiber and effective pore size measurements (*n* = 30–50) denoted as scatter points in each bar graph. **i** Coefficient of variation [%] of the calculated dimensional metrics for each substrate under investigation. **j** Upper part: sample categorization based on their physical dimensionality and fiber architecture: **a** Controls (glass coverslip) as “2D uniform”, “SES-1 min” as “2D random”, “SES-3 min” as “2D less random”, “MEW|0–90°” as “3D uniform” and “MEW|0–45°” as “3D uniform”. Lower part: hypothesized corresponding confinement states induced by each substrate based on representative cell morphologies

The geometrical features of the randomly oriented meshes from SES and the precision-stacked fibrous substrates obtained by way of MEW are quantitatively characterized using scanning electron microscopy (SEM) and stereo microscopy (SM) followed by image analysis with respect to the fiber diameter and the effective pore size distributions (Fig. 3a–d). SES meshes spun for 1 and 3 min exhibit mean fiber diameters of 1 and 0.8 μm, respectively (Fig. 3e). The fiber diameter variation is observed when high-magnification SEM images that focus on small areas (20 × 20 μm) of the fiber mesh like the insets in Fig. 2a, b are reported. However, the morphological characterization results using SEM images across the whole sample area (25 × 25 mm) demonstrate a standard deviation of fiber diameter around ±0.5 μm (Fig. 3e) corresponding to a coefficient of variation (CV) equal to 20–30% (Fig. 3i). These results are consistent with other published studies of optimized electrospinning processes using either PCL or other polymeric material systems^38,39^.

This similarity of mean values of the fiber diameters for the two cases is expected since the material formulation and the process parameters are maintained and only the spinning time is altered. However, the effects of the spinning time on the pore size distributions of the fibrous topographies diverge for SES-1 vs. SES-3. The SES-1 min mesh exhibits significantly greater pore sizes and size variance in comparison to that of the SES-3 min substrate (Fig. 3f), commensurate with the increment in thickness of the randomly oriented fibers deposited continuously on top of each other with increasing processing time.

The fiber diameters and the pore size distributions of the MEW meshes are shown in Fig. 3g, h. The mean fiber diameter is maintained at 14 μm for the two types of precisely-stacked MEW meshes (Fig. 3g). The MEW|0–45° meshes exhibit approximately four times smaller effective pore sizes compared to the MEW|0–90° meshes (Fig. 3h). This is because two additional layers at relative angle offsets of 45° are deposited between the perpendicular fiber layers for the MEW|0–45° meshes while the inter-fiber distance is maintained for both substrates.

The CV of pore size area and fiber diameter is computed for all substrates and plotted in Fig. 3i. Using this metric allows us to categorize all samples and conceptually depict the main goal of the following part of this study, which is to identify if the different type of substrates can yield distinct biological cell confinement conditions (Fig. 3j). This is achieved by modeling single cell confinement states and using them to design a classifier and testing its classification accuracy.

### Effects of substrate architecture on cell confinement

To characterize the effects of the substrate geometry on cell confinement states, neonatal human dermal fibroblasts (NHDFs) are seeded directly on flat glass surfaces (to serve as controls) as well as on solution electrospun substrates (SES-1 min and SES-3 min) and the precision-stacked microarchitectures *(*MEW|0–90° and MEW|0–45°). The details of the cell culturing procedures are provided in the Materials and methods section. The shapes of the fibroblasts are characterized at 24 h after seeding.

**Fig. 4:**
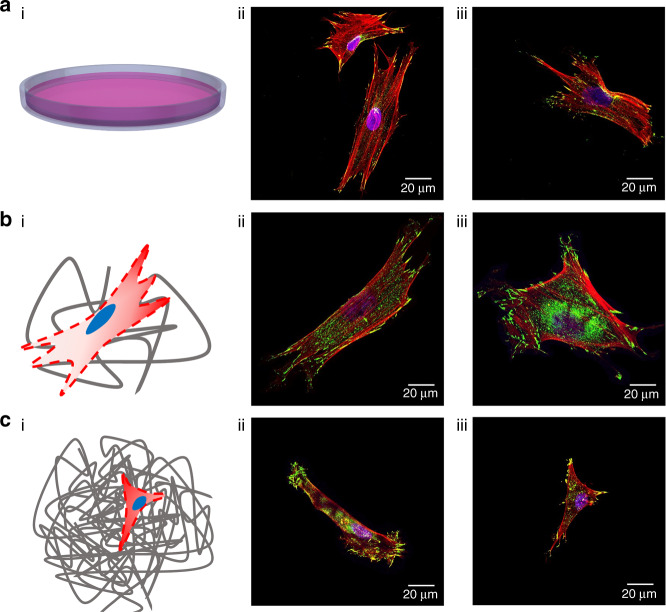
Cell morphology of representative neonatal human dermal fibroblasts (NHDFs) for each mesh. NHDFs are stained for vinculin (green), actin microfilament (red), and nucleus (blue) 1 day after seeding. **a**(i) Concept drawing illustrating attached cells on glass coverslips used as a control culture substrate. **a**(ii–iii) Immunofluorescent images of representative cell morphologies obtained for the control culture substrate. **b**(i) Concept drawing illustrating attached cells on the SES-1 min culture substrate. **b**(ii–iii) Immunofluorescent images of representative cell morphologies obtained for the SES-1 min substrate. **c**(i) Concept drawing illustrating attached cells on the SES-3 min culture substrate. **c**(ii–iii) Immunofluorescent images of representative cell morphologies obtained for the SES-3 min culture substrate. 3D renderings of all depicted cell morphologies are provided in Supplemental Information

It is observed that the cells seeded directly on the flat glass surfaces develop typical fibroblast morphologies exhibiting elongated shapes and distinct actin-based motility structures (Fig. 4a)^40^. It is expected that the cells seeded on the SES-1 min would develop a wider distribution of well-spread lamellar morphologies and this is indeed observed (Fig. 4a). The cells are seen to be at different motility stages at the 24-h mark after seeding. The adoption of variant cell morphologies that are observed in Fig. 4b can be attributed to the non-uniform coverage of the glass substrate by the fibers. There are significant differences at the islands at which fibers are not deposited vs. areas covered by fibers. Cells associated with the SES-1 min substrate are observed to develop punctate vinculin-rich adhesion sites, known as FAs^41^ as shown in Fig. 4b, respectively. The observed FAs are distributed throughout the cell body forming an elongated shape at the end of branched cellular protrusions that extend the broad actin-rich lamellipodia that are ribbon-like broad flat cellular protrusions formed at the leading edge of a migrating cell^42^. This is a typical morphological characteristic observed in cells cultured on flat surfaces (glass or plastic)^15^. In contrast, cells seeded on SES-3 min demonstrate significantly smaller spreading with less actin stress fibers traversing the cytoplasm and micro-spikes that protrude marginally beyond the cell front and rear edge and are composed of actin bundles together with FAs (Fig. 4c). Lastly, a common characteristic that is observed with cells seeded on flat glass surfaces and SES substrates is the development of an actin-enriched lamellipodium.

**Fig. 5:**
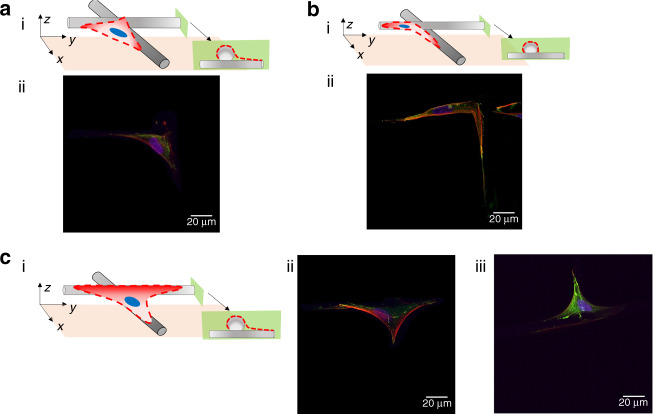
Cell morphology of representative neonatal human dermal fibroblasts (NHDFs) at distinct locations on the MEW|0–90° culture substrate. NHDFs are stained for vinculin (green), actin microfilament (red), and nucleus (blue) 1 day after seeding. **a**(i) Concept drawing illustrating an attached cell at a specific location within the 3D mesh. **a**(ii) Immunofluorescent image of the previously illustrated attached cell. **b**(i) Concept drawing illustrating an attached cell at a specific location within the 3D mesh. **b**(ii) Immunofluorescent images of the previously illustrated attached cell. **c**(i) Concept drawing illustrating an attached cell at a specific location within the 3D mesh. **c**(ii–iii) Immunofluorescent images of the previously illustrated attached cell

It is observed that the cells seeded on MEW|0–90° are mainly attached along single fibers and at the intersection of layered fibers. In the former case, cells adopt thin elongated shapes dictated by the curvature of the fiber, since they “grab” the exposed areas of the fiber at different planes (Fig. 5a–c). In the latter case, cells adopt uniform shapes and demonstrate spreading, the degree of which depends on the number of fibers at the intersection point.

**Fig. 6:**
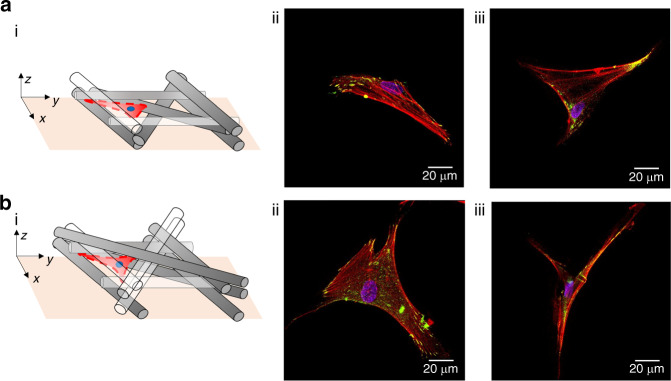
Cell morphology of representative neonatal human dermal fibroblasts (NHDFs) at different locations on the MEW|0–45° culture substrate. NHDFs are stained for vinculin (green), actin microfilament (red), and nucleus (blue) 1 day after seeding. **a**(i) Concept drawing illustrating an attached cell at a specific location within the 3D mesh. **a**(ii–iii) Immunofluorescent image of the previously illustrated attached cell. **b**(i) Concept drawing illustrating an attached cell at a specific location within the 3D mesh. **b**(ii–iii) Immunofluorescent images of the previously illustrated attached cell. **c**(i) Concept drawing illustrating an attached cell at a specific location within the 3D mesh. **c**(ii–iii) Immunofluorescent images of the previously illustrated attached cell. 3D renderings of all depicted cell morphologies are provided in Supplemental Information

Cells seeded on MEW|0–45° are confined and suspended at various levels across the thickness of the substrate and within the porous microenvironments defined by layered fibers (Fig. 6a, b). All imaged cells develop triangular lamellar shapes consistent with the enforcing triangular microarchitecture of the substrate. The cell shapes are characterized by relative few actin stress fibers that traverse the cytoplasm and terminate in distinct filopodia, with elongated FAs sequestered to the tips of the protrusions.

Taken together, these findings qualitatively suggest that randomly stacked (SES) and precisely-stacked (MEW) fibrous substrates exhibit topographies that are different and the resulting cell morphologies depend on the geometry of the topography of the substrate. Different structures impose different cell confinement states. First, the cellular and subcellular morphological features of cells seeded on SES and MEW substrates give rise to different confinement states that are different with respect to the ones observed in the unconfined cells cultured on glass coverslips. Second, there exist important qualitative differences in cell shapes and FA distributions, dependent on whether randomly-oriented solution electrospun mesh substrates or the precision-stacked woven mesh substrates fabricated via the MEW process are used.

### Machine learning-based metrology

#### Image-based feature extraction

**Fig. 7:**
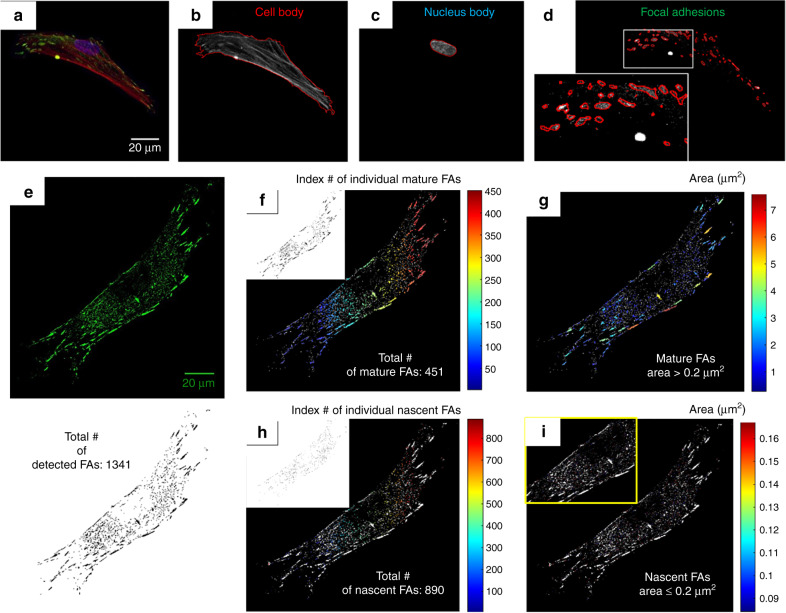
Single-cell bioimage analysis for feature extraction. **a–d** Performance demonstration of the proposed automated image processing algorithmic workflow using a representative cell cultured in the MEW|0–45° and SES-1 min for panels (**a**–**d**) and (**e**–**h**), respectively. **a** Colorized multi-channel maximum projection image obtained by combining three different single channel maximum projections. Single channel maximum projections are obtained by processing the Z-stack raw images. Red channel: cytoskeleton, blue channel: nucleus, green channel: vinculin. **b** Grayscale maximum projection of the red channel image overlaid with the contour of the segmented cell body. **c** Grayscale maximum projection of the blue channel image overlaid with the contour of the segmented nucleus. **d** Grayscale maximum projection of the green channel image overlaid with the contour of the segmented FAs. **e** Maximum projection of the green channel image of a representative cell cultured on SES-1 min. **f**–**i** Performance demonstration of the proposed automated image processing algorithmic workflow for mature (**f**, **g**) and nascent (**h**, **i**) FA detection and segmentation based on individual FA area ([μm^2^], SI). The images in (**f**) and (**h**) are overlaid with the contour of all the segmented adhesion sites colorized based on their index number

The imaged cell shapes are modeled using machine-learning frameworks. The aim is to quantitatively characterize and classify the observed multiscale morphological differences. The details of the machine learning framework are described in the Experimental section. In this framework, the single cell maximum intensity projections of each fluorescent channel (red, green, blue) are employed to detect the cellular (cytoskeleton) and sub-cellular features of interest (FAs, nucleus). An algorithmic workflow of image processing tasks is developed to provide the segmentation and subsequent morphometric and distribution analysis of these features. The image-based feature extraction procedure is described in detail in the Material and methods section. Typical outcomes of this procedure are depicted in Fig. 7. The figure shows the colorized max projection of a representative cell of the 3D microscale fibrous substrates (Fig. 7a) along with its raw grayscale channel images. Each channel image is overlaid with contours of the following segmented features of interest: (a) the cell body (Fig. 7b), (b) the nuclei body (Fig. 7c), and (c) mature FAs (Fig. 7d).

Of importance is the rationale behind the term “mature FAs” and their automatic sorting from the initially-segmented, vinculin-rich adhesion sites within each imaged cell. Adhesions have been previously classified into nascent adhesions, focal complexes and FAs based on their size (~0.1–10 μm^2^) and localization within the cell body^41^. It is known that during FA maturation, nascent adhesions assemble soon after the integrin receptors engage with the extracellular matrix (ECM) at the edge of the lamellipodium^41^. At this point, they are either undergoing fast turnover during active protrusions or are evolving into focal complexes within the lamellipodial dendritic actin network^41^. These adhesions grow and elongate into FAs connected by bundles of actin stress fibers at the lamellipodium–lamella interface^41^. Masks of all the adhesion sites detected in a representative cell from the control substrate are illustrated in Fig. 7e.

In the present study, the detected adhesion sites are sorted into two bins based on their surface area (below or above 0.2 μm^2^) following previous experimental FA characterization studies in mesenchymal stem cells (MSCs) cultured on flat substrates^43^. MSCs and the fibroblasts are considered to be similar cell model types for adhesion studies^20^. The following categorization is adopted in the present study. Detected adhesion sites with individual surface areas that are smaller than 0.1 μm^2^ are considered as cytosolic background and are excluded from the analysis. Nascent adhesions are those with an individual surface area that is smaller than 0.2 μm^2^ (Fig. 7g). Adhesions with an individual size larger or equal to 0.2 μm^2^ are considered to be mature FAs (focal complexes and FAs). It is the mature FAs that are the subject of our metrology studies (Fig. 7h) the results of which are presented next.

#### Morphometric analysis

**Fig. 8:**
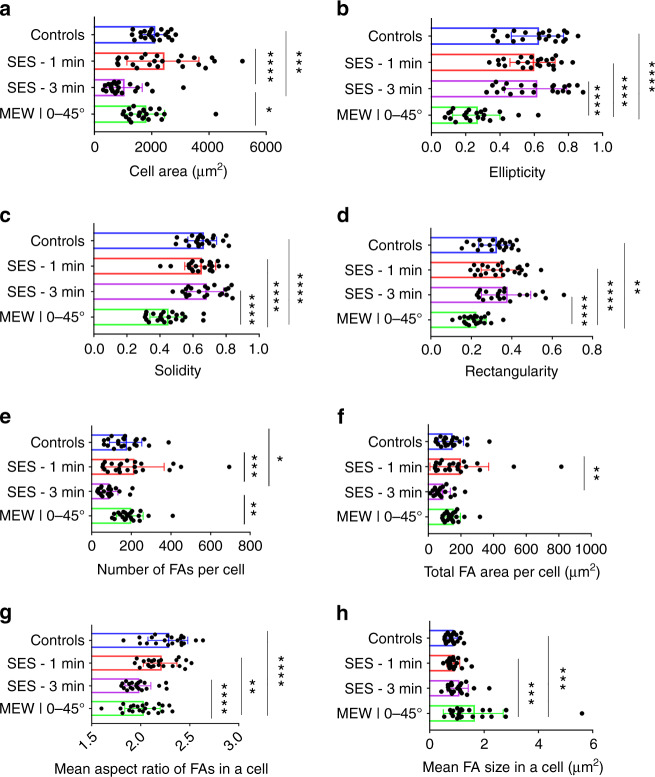
Morphometric profiling results. **a**–**d** Size and shape-related metrics of the detected cell bodies. **e**–**h** Size and shape-related metrics of the detected FAs; mean ± standard deviation, total of *n* = 88 cells analyzed, *P ≤ 0.05, **P ≤ 0.01, ***P ≤ 0.001, ****P ≤ 0.0001

Typical metrology results obtained for describing the statistics of cell size and shape are shown in Fig. 8. As expected, cell areas computed for cells cultured on the SES-1 min substrate demonstrate substantial variance (Fig. 8a). This can be explained by the substrate’s high degree of topographical heterogeneity that is consistent with the relatively high CV of its pore sizes. Cells cultured on SES-3 min substrates demonstrate the smallest cell area across all analyzed groups. This is in line with previous observations of characteristic cell morphologies in Section B. The remaining cell population groups do not demonstrate any statistical significance with respect to the mean cell area despite the topographically different underlying substrates. However, the negative statistical results indicate a high statistical probability that cells cultured on the 3D microscale fibrous substrates (MEW|0–45°) will not reside on the 14 μm fibers (leading to smaller cell areas), but rather migrate inwards through the pores to assume a suspended state. This expectation is in line with the previous observations of representative cells (Fig. 6) assuming a triangular shape with different orientations and attachment points at various levels across the thickness of the substrate.

Cellular shape descriptors based on moment invariants are directly accessible via the MIPAR software (Fig. 8). Such descriptors reveal global cell shape differences across the cell population groups^44,45^. The MEW|0–45° cell population group demonstrates the smallest mean ellipticity (Fig. 8b) and rectangularity (Fig. 8d), as expected based on initial observation of the cell shapes depicted in Figs. 4 and 6. It is evident here that all cells seeded on MEW substrates exhibit a triangular cell shape that will yield smaller rectangularity values compared to cells seeded on flat and SES meshes that are elliptical and more rectangular in nature. The small standard deviations obtained for the MEW|0–45° cell population with respect to both metrics are indicative of relatively high cell shape homogeneity associated with the homogeneous structure of the MEW woven meshes. This is further reinforced based on the understanding that most of the cultured cells reside at the triangular intersections of the porous microenvironments provided by the MEW|0–45° substrate. Lastly, solidity is employed as a representative metric with the triangular and concave observed in the MEW|0–45° substrate demonstrating smaller solidity values compared to the more elliptic cells across the controls and the electrospun (SES) fibrous substrates (Fig. 8c).

The second part of the morphometric analysis focuses on the statistics for the detected mature FAs. Similar to the observations for cell area, the SES-3 min group demonstrates the smallest mean FA number (Fig. 8e) across all cell population groups implying that the number of mature FAs correlates with the degree of cell spreading. Furthermore, the level of mature FAs coverage appears to be the same across all groups with the SES-3 min group demonstrating the smallest one (Fig. 8f). FA shape is quantified based on the FA aspect ratio metric. The controls and the MEW|0–45° group exhibit the highest and lowest aspect ratios, respectively, i.e., the most and least elongated mature FAs, respectively (Fig. 8f). The degree of individual FA elongation correlates positively with the ellipticity of the global cell shape (Fig. 8g) verifying the shape-bearing role of mature FAs. On the other hand, the mean individual FA size metric demonstrates an inverse pattern with the MEW|0–45° group demonstrating the highest mean value (Fig. 8h).

#### FA distribution analysis

**Fig. 9:**
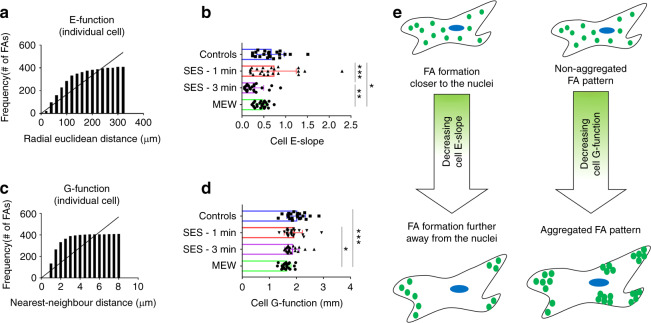
Distribution analysis results. **a** Cumulative frequency distribution graph plotting the frequency of mature FAs detected within an individual cell as a function of the respective radial Euclidean distances computed based on the detected nucleus and FAs centroids. **b** Cell E-slope; mean ± standard deviation, total of *n* = 88 cells analyzed, *P ≤ 0.05, **P ≤ 0.01, ***P ≤ 0.001, ****P ≤ 0.0001. **c** Cumulative frequency distribution graph plotting the frequency of mature FAs detected within an individual cell as a function of the respective nearest neighbor distances computed based on the detected FAs centroids. This is defined as the G-function. **d** Cell G-function metric; mean ± standard deviation, total of *n* = 88 cells analyzed, *P ≤ 0.05, **P ≤ 0.01, ***P ≤ 0.001, ****P ≤ 0.0001. **e** Visual illustration of the spatial distribution metrics for mature FAs

It is then tested, whether the cell population groups demonstrate any differences concerning the spatial distribution of mature FAs. The relative location of individual mature FAs with respect to the nuclei centroid (E-function) and their nearest neighbor (G-function) are plotted at the single cell level using cumulative frequency distribution functions (Fig. 9a, c). Based on the definition of these spatial distribution metrics, mean metrics are computed to identify differences at the cell population level (see Materials and methods for a detailed explanation of these metrics). The results indicate that the MEW|0–45° substrate is characterized by cells that tend to develop more clustered mature FAs (Fig. 9d, e) with the higher number of them being closer to the nucleus (Fig. 9b, e) compared to cells residing on flat surfaces (controls). This is consistent with the observed cells being suspended and attached at distinct points across the 3D MEW|0–45° substrates, as opposed to cells on the flat surfaces or on randomly oriented electrospun meshes, where the cell–substrate contact interface is larger resulting in the presence of mature FAs formation across the whole cell body (Fig. 9e).

#### Learning and classifying cell shape phenotypes

**Fig. 10:**
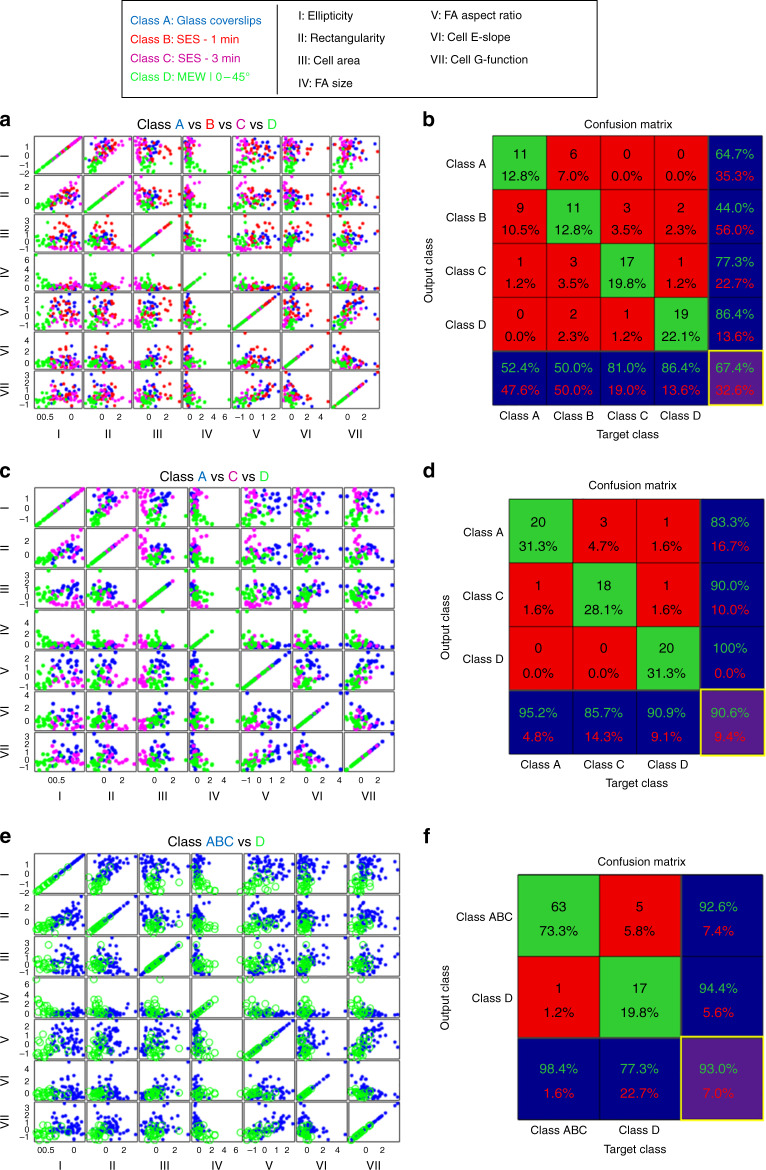
Classifier design and accuracy evaluation results for 3 different classification tasks. Support vector machines are used for all the machine learning tasks. **a** Multi-class classification task taking into consideration all classes. Scatter plot for the combination of metrics. Blue, red, pink, and green points represent processed cells from Class A, B, C, and D, respectively. 88 cells are processed whereby the computed are normalized and then plotted to assess the classifier design results. **b** Confusion matrix with average classification accuracy outlined with yellow color (64.4%). **c** Multi-class classification task taking into consideration only Class A, B, and C. **d** Confusion matrix with average classification accuracy outlined with yellow color (90.6%). **e** Binary classification task by combining Classes A, B, and C in one class designated as Class ABC against Class D. **f** Confusion matrix with average classification accuracy outlined with yellow color (93%)

While the initial assessment of the discriminatory information of each metric provides valuable insights concerning the cell shape phenotypic differences across and within each cell population group, the ability to infer the substrate dimensionality and architecture directly from single cell morphologies remains to be validated. To accomplish that, the single-cell multi-dimensional data sets are used to train a machine learning algorithm (see Materials & Methods section) with the aim of distinguishing between four different classes by considering all features simultaneously. The seven features computed during the metrology part and used for the machine learning tasks in this section are defined on the basis of non-interdependence with respect to computing (there isn’t any feature that is needed to compute another feature) and biological relevance. The class declaration is depicted in the legend of Fig. 10, where all substrate dimensionalities and topographies are depicted along with the cell confinement states that were previously implied. Three different classification tasks are performed. Combinations of the scaled metrics are plotted to allow easier assessment of the results (Fig. 10a, d, g). The capability of the classifier to operate satisfactorily with data outside the training set for each classification task is assessed based on the classification accuracy (Fig. 10c, f, k).

Initially, the multi-class classification problem is attempted by taking into account cell morphologies across all the fabricated substrates (Fig. 10a). The classifier demonstrates a low classification accuracy (67%), which can be explained by the large intra-class variance of Class B (Fig. 10b). It is important to mention that among all four different classes, Class D has a higher classification accuracy since the features of Class D have a separable distribution with tighter variance from the features of classes A, B, and C (also evident in Fig. 10a, b) (Fig. 10c). By removing Class B, the classification accuracy increases to 90.6%, demonstrating that the trained classifier can predict with high accuracy the substrate from which a cell originates based strictly on its feature vector identity. Remarkably, when the binary classification task is run by combining all classes corresponding to the flat or electrospun SES substrates, including the “noisy” Class B against Class D, the classification accuracy level remains around 93%. Thus, it is demonstrated that the 3D microscale precision-stacked substrates promote a confined and suspended state that morphologically stands out both at the cellular as well as the sub-cellular FA level.

#### Cell shape heterogeneity in fibrous substrates

Lastly, the substrate structural heterogeneity with respect to fiber diameter and pore size distribution dictates the variance of the defined morphometric and protein distribution metrics with the MEW|0–45° and SES-3 min substrate demonstrating the most and least homogeneous population of single cell morphologies, respectively.

**Fig. 11:**
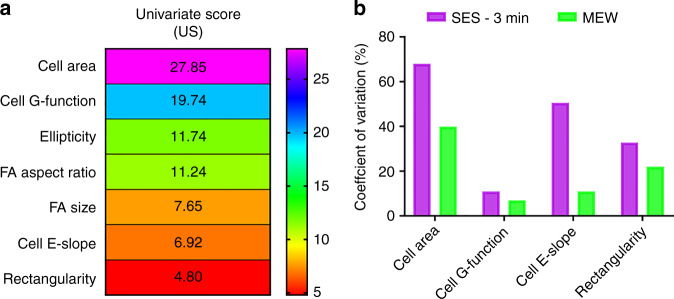
Statistical testing for cell shape heterogeneity in SES-3 min substrates vs. MEW substrates. **a** Univariate feature selection results in descending order obtained from one-way ANOVA *F*-tests. Univariate score (US) is equal to the ratio of the variance across the two different classes to the variance within class. The feature demonstrating the highest and the lowest US values are the most and the least significant for the classification task performed by the SVM machine learning algorithm. **b** Coefficient of variation [%] of the two most and the two least significant features based on the univariate scoring results

To provide a quantitative estimate of MEW heterogeneity vs. SES heterogeneity, a univariate feature selection implemented that can inform which features were used by the classifier to separate Class C (SES-3 min) from Class D (MEW-0–45°) during the first classification task A vs. B vs. C vs. D (Fig. 10a–c). The results demonstrate that the most significant features whose variation across classes is higher relative to their variation within each class are the following: “cell area”, “cell G-function” and the least significant ones, whose variation within each class is higher relative to their variation across classes are the following: “cell E-slope”, “rectangularity”. Reporting only the most important features might exclude other features that did not play an important role during the multi-class classification task (A vs. B vs. C vs. D) but whose heterogeneity is of significant biological importance. As a last step, the CV [%] of the two most and least significant features is computed and plotted in Fig. 11. The results demonstrate that the MEW substrates provide tighter control over both the most and the least significant features that the SVM machine learning algorithm used for the classification task.

## Discussion

Although the modulation of cellular phenotype with biochemical regulatory factors is well-known, structural and mechanical inputs from the ECM have been identified as key regulators of measurable cell phenotypic attributes. To investigate the effects of the physical properties of the matrix microenvironment on cellular phenotype, microfabrication technologies and 3D cell encapsulation technologies have enabled the identification of previously ignored structural and dimensional parameters, respectively, that are crucial for precisely engineered biomaterial substrates.

In order to independently modulate these substrate parameters within a coherent experimental model, we have demonstrated the marriage between electrospinning and additive manufacturing towards the 3D fabrication of high-fidelity biomaterial fibrous substrates with geometrical feature sizes at cell operating length scales. Furthermore, we have advanced a machine learning-based metrology framework that can quantitatively assess and classify the effect of geometrical confinement on human adherent cells across different fibrous substrates dimensionalities and architectures. To measure this effect, we have demonstrated a quantitative confocal imaging workflow that reveals distinct confinement states both at the cellular and subcellular FA protein level. The classification results demonstrate that cells assume distinct confinement states that are enforced by the prescribed substrate dimensionalities and porous microarchitectures.

It is noteworthy to mention that the reproducibility and biological relevance of the advanced system may be further augmented by coating the fibrous substrates with ECM proteins (fibronectin, vitronectin, collagen)^46–48^ or a conjugated RGD-peptide used in PEG-hydrogels^49^. To be sure, the poly-l-lysine (PLL) prescribed in this study to promote cell attachment in an integrin-independent manner could affect the overall metrology described herein. Therefore, further experimentation would serve to validate whether the programming of downstream cell morphology with precision substrate geometry design parameterization may be similarly observed using native ECMs that promote integrin-dependent attachment to the substrates. Lastly, de novo production of ECM proteins may also play a role in adhesion organization and possibly diverge from the metrology results reported here. The latter was not tested experimentally due to the short time course examined between cell seeding and screening (24 h post-seeding), which, according to previous fibroblast culture studies represents a smaller time window compared to that required before the earliest appearance of collagen formation^50^.

In the context of our study, the demonstrated PCL substrate material system is not advanced with the intention to replace biological gels for studying mechanosensing in an in vivo context. Based on the specific aim of this paper to fabricate substrates where precision geometries can be reproduced and isolated as an independent variable and tested with seeded cells, the MEW substrate offers a highly controllable 3D system with respect to porous microarchitecture at cellular-relevant length scales. The metrology and classification results show that there is a tight link between the porous architecture and the induced cell shape phenotypes. Using these substrates, cell biologists could study mechanotransduction phenomena for different cell shapes that are induced geometrically in a 3D environment where ECM remodeling (fibrous architecture variation as cells migrate) is decoupled from resultant local stiffness variations. The use of simpler biomaterial systems with tight control over certain characteristics may help understand which characteristics of more complex systems such as biological gels are important for proper mechanosensing in vivo.

We have established a technology platform that serves as a major step towards the development of bioinformatics-guided additive manufacturing systems, one that promises insight into cellular interactions beyond the reach of current phenotypic control and analysis. The combination of advanced fabrication and metrology tools paves a new avenue for the systematic engineering of functional biomaterial systems that can reliably guide distinct, uniform, and predictable cell responses for a wide range of biomedical applications. The need for tighter control over cell function is a major roadblock for getting tissue engineering products to the clinic^42^. Currently, the noise in cell phenotype makes it harder to detect positive outcomes during a clinical trial. Therefore, any measures taken to tighten specifications on the substrate, and thereby also tighten the variance in cell phenotype, is much needed by this industry^51^.

For example, we have preliminarily shown that there exists an operational window of geometrical parameters attributed to an ordered fiber-based material matrix substrate that map to unique states of biophysical cell confinement characterized by homogeneous cell shape phenotypes. Therefore, we expect that there exists granularity in the geometric confinement states that will yield the phenotypic spectrum of whole cell and subcellular morphometric features, along with different functional outcomes for various model cell types, including differentiation in stem cells. The range and sensitivity of this operational parameter space will determine the extent to which cellular phenotype can be controlled. Advancing a technology platform that leverages a shape-driven control pathway to create and maintain a desired phenotype at the single cell and population level is potentially far-reaching for fundamental cell biology and regenerative medicine, respectively.

## Materials and methods

### Biomaterial substrate

Pure PCL (Capa6500) pellets (with number average molecular weight of 45,600 g/mol and polydispersity of 1.219) that are obtained from Perstorp UK Ltd. (UK) is the biomaterial substrate that was used for the experimental study in this paper. PCL has been extensively used in the biomaterials field due to its biocompatibility, long-term biodegradability, and relatively low and wide melt processing range (60–90 °C). Furthermore, PCL’s tunable viscoelastic properties make it amenable for melt extrusion-based additive manufacturing techniques towards the fabrication of scaffolds for tissue engineering applications^52^.

### Non-woven fibrous substrate fabrication

A custom-built SES apparatus is employed for the fabrication of two-dimensional (2D) non-woven PCL microfibrous meshes. A volume of 5 mL PCL working solution with 12% [w/v] final concentration is prepared by mixing PCL pellets with HFIP by gentle overnight magnetic stirring at 1000 RPM. A stainless-steel needle tip (21 gauge) with blunt end is attached on a plastic Luer-lock syringe (3 mL). 1 mL of final PCL working solution is slowly loaded in the syringe barrel without introducing any bubbles. The loaded syringe is clamped on a programmable syringe pump (Harvard Apparatus). The positive lead of a high DC voltage power supply is attached on the needle tip and the ground lead on an aluminum collector vertically placed at a distance equal to 12–13 cm. PLL coated glass coverslips are taped on the grounded aluminum collector. Non-woven fibrous meshes are collected on the glass coverslips at a volumetric flow (*Q*) rate equal to 10 μL/min (*Q* = 10 μL/min) and voltage potential (Vp) equal to 15 (Vp = 15 kV) for different spinning times. Samples obtained for spinning time equal to 1 min and 3 min are designated as “SES-1 min” and “SES-3 min”, respectively.

### Woven fibrous substrate fabrication

A custom-built high-resolution additive manufacturing system, whose operating principle is based on the direct writing of melt electrospun fibers (also known as “melt electrospinning writing—MEW”) is employed for the fabrication of 3D woven PCL microfibrous meshes. The design and experimental modeling of the established MEW system configuration has been previously described in detail^37^. Prior to printing, PCL pellets are loaded in a glass Luer-lock syringe that is vertically placed into a vacuum convective heat oven overnight to remove any bubbles that may affect the process stability and downstream structural formability of the melt electrospun fibers. After assuring the temperature homogeneity of the polymer melt, a stainless-steel needle tip at a prescribed diameter (Dt = 21 G) is adapted onto the syringe. The syringe with the attached tip is then placed in the material head of the system, which is kept at 78 °C at a melting temperature (Tm) equal to 78 °C. The volumetric flow rate (*Q*) is set and controlled at 25 μL/h using a programmable syringe pump (Harvard Apparatus, USA). The voltage potential (Vp) between the needle tip and a grounded aluminum collector is set equal between 10 and 12 kV (10 kV ≤ Vp ≤ 12 kV) using a high DC voltage source (Gamma, USA). The aluminum collector is mounted on a x–y programmable stage (ASI Applied Scientific Instrumentation, USA) with high positional accuracy (~10 μm) at a wide dynamic range of translational speeds (1 < *U*_T_ < 80 mm/s). Custom translational patterns are written in Python 2.7 guiding the stage to move at various patterns and speeds. Rectangular microscope glass coverslips (25 × 25 mm and thickness range: 0.13–0.17 mm—Fisher Scientific, USA) are attached on the aluminum grounded collector and used as the collection substrates of the MEW fibers. The TCD (*d*) is set equal to 12 mm (*d* = 12 mm). 3D woven fibrous substrates (10 layers of fiber and 100 fibers/layer) with uniform fiber diameter and two different lattice microarchitectures are fabricated in a layer by layer manner by controlling the inter-fiber distance and relative fiber deposition angle for the prescribed set of process parameters. Samples with 90° and 45° inter-layer fiber orientation are designated as “MEW|0–90°” and “MEW|0–45°”, respectively.

### Fibrous substrate characterization

SES-1 min and SES-3 min samples are examined using SEM. MEW|0–90° and MEW|0–45° are examined using SM. The structural formability of all the fibrous substrates is quantitatively characterized with respect to fiber diameter and effective pore size area. The fiber diameter is measured directly from the acquired micrographs by randomly sampling regions (*n* = 100) across three replicates of each type of fibrous substrate for statistical significance using Fiji software^53^. The apparent pore size distributions were measured directly from the acquired micrographs using the MIPAR image processing software^54^. A custom semi-automatic segmentation recipe was developed based on contrast and brightness preprocessing steps, the application of classical thresholding algorithms (based on the grayscale intensity difference between the background and the printed fibers), and the subsequent manual correction of erroneous segmented areas. The mean fiber diameter and mean effective pore size are reported along with their standard deviation for each type of fibrous substrate under investigation.

### Biological materials

NHDFs (Coriell Institute) were cultured in high-glucose Dulbecco’s modified Eagle’s medium (DMEM) containing 1% penicillin/streptomycin and 10% fetal bovine serum in basal media. Rectangular microscope glass coverslips (25 × 25 mm and thickness range: 0.13–0.17 mm—Fisher Scientific, USA) and the fabricated PCL meshes with prescribed feature sizes are implemented as the control and experimental substrates, respectively. Both substrates were placed inside sterilized, non-treated 6-well plates for all biological studies. Both groups of substrates are seeded with NHDFs at the P8 generation. NHDFs are cultured on the different substrates for 24 h at incubating conditions (37 °C, 5% CO_2_). Cell seeding densities were kept at 2000 cells/coverslip (2D controls) and 5000 cells/fibrous substrates (3D) to allow single cell morphology observation. Prior to seeding, both controls and fibrous substrates are placed inside Petri dishes, sterilized with 70% ethanol, dried under the exposure of UV light for at least 1 h, and then transferred to sterile 6-well plates where they were coated with sterile filtered PLL (0.01%, P4707 Sigma Aldrich, USA) to promote cell attachment according to manufacturer’s protocol. Specifically, 0.5 mL of that solution is aseptically transferred to all substrates. After 5 min in room temperature, the excess solution is removed, and the surface is thoroughly rinsed with sterile water and allowed to dry for at least 2 h inside the biosafety cabinet hood. The coated substrates are then exposed to UV light overnight for sterilization. Right before cell seeding the substrates are thoroughly rinsed with final media formulation. Cell morphology is observed using immunofluorescent staining at day 1 (24 h after cell seeding).

### Immunofluorescent staining

Attached NHDFs are fixed in 4% paraformaldehyde (in PBS) for 5 min at room temperature, permeabilized with 0.2% (v/v) Triton-X and blocked with 4% BSA. To examine FA distribution, samples are incubated with primary antibodies (vinculin: 1:200 mouse monoclonal antibody (Abcam)) and secondary antibodies (1:200, Alexa Fluor 488 donkey anti-mouse IgG (H+L) (Abcam)). To examine actin cytoskeletal organization, the samples are stained with Texas Red-X phalloidin (1:400, Invitrogen). Prior to imaging, droplets of Fluoroshield mounting medium with DAPI (0.0002%, Abcam) are applied to the samples to allow cell nuclei observation and to prevent photobleaching. Excess medium is then removed by touching the edges of the slide against a paper towel. The samples are set to stand at room temperature for about 5 min and a coverslip is carefully on top of them avoiding air bubbles. The edges of the coverslip are sealed with nail polish to avoid the formation of bubbles over time. The majority of all samples were imaged directly after the immunostaining procedure is done or within 24 h during which there were stored in the dark at 2–8 °C.

### Quantitative confocal microscopy

3D confocal microscopy raw data are employed to detect cellular (actin microfilaments) and sub-cellular morphometric features (FAs, nuclei) using image processing. The analysis followed for detecting and quantifying the features of interest is performed with the open source software Fiji^53^ and MIPAR software^54^. Samples are imaged at ×40 magnification on an inverted motorized microscope (IX83 Olympus, USA). The samples are also imaged with a confocal laser-scanning microscope (Zeiss LSM 510) equipped with a ×63 oil immersion objective. The samples are scanned across their thickness with a step size of 0.1 μm. Z-stack images with 488-, 543-, and 633-nm laser wavelengths were acquired corresponding to the green, red, and blue channels, respectively. Raw Z-stack images are post-processed using the ImageJ software and, unless otherwise specified, are presented as maximum intensity projections.

### Image-based cell feature extraction procedure

The analysis followed for detecting and quantifying the features of interest is performed with the open source software Fiji^53^ and the beta version of the MIPAR software^54^. A fully automated procedure was developed to determine cell body and nuclei contours using the red and blue fluorescent channel images, respectively. In this procedure, fluorescent images are transformed to 8-bit grayscale images and pre-processed to ease the automated segmentation procedure, explained hereafter. Initially, brightness and contrast are equalized across the image by performing a uniform histogram scaling using the Contrast Limited Adaptive Histogram Equalization (CLAHE) algorithm^55^. Then, the image is denoised using an advanced filtering algorithm known as Non-Local Means (NLM)^56^. During that step, each pixel’s local window is compared to windows around it and then the windows’ center pixels are averaged together with weights depending on the variations between the windows. This step is crucial for the accuracy and objectivity of the segmentation since it allows for noise reduction while preserving the edges of the features of interest. The phalloidin and DAPI signals are mostly present on the actin microfilaments and on the border of the nuclei, respectively. Thus, segmentation using thresholding results in incomplete cell body and nucleus mask, in which the center is not filled and the border is not continuous. Following the NML step, segmentation is performed using thresholding during which the image is binarized based on a certain pixel threshold value. The threshold value is obtained automatically using Otsu’s method^57^. Despite the effectiveness of the NLM step into preserving the borders of the segmented feature, high gradient values in the cytoskeleton or the nucleus caused by non-homogeneous content, require an additional erosion step. During that step, black pixels are removed if they are surrounded by white pixels, whose number is greater than or equal of a user-specified value. It was determined that a value of 5 was suitable for all the images. The binarized image is inverted resulting in an image with white background and a black mask of the feature of interest. The algorithmic workflow is completed by adding an additional “hole filling” step that ensures the removal of any redundant white pixel features that might cause discontinuities within the black mask of the segmented features of interest. The detection and segmentation of FAs is performed using the same algorithmic workflow with the addition of some extras filtering steps that allowed the removal of noisy signal due to cytoplasmic background and the isolation of the mature FAs with respect to nascent adhesions. The former one is achieved by adding an extra dilation step before the erosion step. The latter one is achieved by adding an extra filtering step that removes all the black pixel features with an area equal or smaller to 0.2 μm^2^. The image processing workflow is described in detail (Supplemental Document) with critical settings used for each filtering step along with the image outcome after each filtering step.

### Definitions of size-, shape-, and distribution-related metrics

Metrics of the segmented features of interest are defined hereafter. The “Cell Area” metric is defined as the spreading area of individual cells. The “Ellipticity” is defined based on moment invariants^58^ as previously described^45^. The moment invariants are directly obtained from the MIPAR software after the image-based cell feature extraction procedure is completed. The “Ellipticity” metric range over [0,1] peaking at 1 for a perfect ellipse. The “Rectangularity” metric is defined as the ratio of cell’s area against the area of its minimum bounding rectangle as previously described^44^. The MBR area is computed using the length of each feature’s bounding box in the *x* and *y* direction. The lengths in both directions are directly obtained from the MIPAR software after the image-based cell feature extraction procedure is completed. The value of the “Rectangularity” ranges over [0,1] peaking at 1 for a perfect rectangle. “The “Solidity” metric is defined as the ratio of the area of each cell over the area of the tightest fitting convex hull. It takes values between 0 and 1 with the ratio approaching to 1 as the cell area increases to match the fitted convex hull. Thus, solidity is an indicator of how “ruffled” or concave the cell periphery of the cell is. The “FA size” metric is defined as the area of individual mature FAs. FA shape metric is quantified based on the “FA Aspect Ratio”, which is defined as the ratio of the major to the minor axis length of an ellipse fitted into each detected FA.

The Cartesian data of the nuclei and FA masks are leveraged to extract the centroids of the detected nuclei and individual FAs, respectively. Using these data, two functions are defined: (a) the *E-function* and (b) the *G-function*. The *E-function* is defined as the cumulative frequency distribution of the radial Euclidean distance of each FA centroid from the nuclear centroid within each cell. Straight lines constrained on the origin of the Cartesian axes are fitted on the *E-function* curves using linear regression. This procedure incorporates fitted slopes (denoted as “*Cell E-slope*”) as metrics to compare individual cells with respect to the tendency of FAs to form either nearer to or distant from the nuclei. Averaging all the fitted “*Cell E-slopes*” obtained from the fitting of E-functions of cells cultured under identical substrate conditions enables a “*mean E-slope*” value as a metric to compare different cell populations. Moreover, the *G-function* is defined as the distance of each detected FA to its nearest detected FA neighbor. Averaging the distance values within each cell enables a metric denoted as “*Cell G-function*” to compare the degree of FA clustering between individual cells. Averaging the “*Cell G-function*” values obtained for cells cultured under the same substrate conditions, a “*mean G-function*” value can be used as a metric to compare the degree of FA clustering between different cell populations.

### Statistical analysis

Based on the experimental design, the mean difference for each defined metric and between each of the four cell population groups corresponding to the glass coverslip (controls) and the three fibrous substrates (SES-1 min, SES-3 min, and MEW|0–45°) were compared using one-way ANOVA and Tukey’s multiple comparisons tests. The sample size of each group was chosen with respect to the maximum number of individual cells that can be imaged efficiently on each substrate using confocal microscopy (*n* = 20–22 cells/group). Two-tail *P*-values with 95% confidence intervals (CI) for the computed mean difference obtained from the Tukey’s multiple comparison tests are considered.

### Classification scheme

A 7-D Cartesian coordinate system of cell shape phenotypes, in which each axis represents each feature metric, is developed for the 7-metrics computed from the various measures of cell shapes, i.e., the “morphometric” analysis and the spatial distributions of FAs. The metrics included (a) Ellipticity (“I”), (b) Rectangularity (“II”), (c) Cell Area (“III”), (d) FA Size (“IV”), (e) FA Aspect Ratio (“V”), (f) E-Slope (“VI”), (g) Mean G-function (“VII”). Within this representation, each point represents one single-cell feature-vector with 7 elements corresponding to the computed metrics for the specific cell. All metrics are normalized using a *Z*-score function, which centers and scales all metric values to have zero mean and unit standard deviation, respectively^59^.

The transformed metric vectors for each cell population are multidimensional data sets to train a support vector machine (SVM) with a linear kernel using the classification learner package in Matlab^60^. The linear-kernel SVM is a supervised machine learning algorithm that can classify the data by determining the best hyperplane that distinguishes all data points into the defined classes^59^. The best hyperplane for the SVM algorithm is considered the one with the largest margin between the two classes with the margin being the maximum width of the slab parallel to the hyperplane that has no interior data points. The predictive accuracy of the linear-kernel SVM is assessed using a 5-fold cross-validation scheme to protect against overfitting and to assure the generalization performance of the classifier^61,62^. Here, the data are randomly partitioned in 5 folds where, for each fold, the scheme trains the linear SVM using the out-of-fold observations and assesses the model performance using the in-fold data. The classification accuracy is defined as the average percentage of the correctly classified data for each fold and used as a metric for the classifier’s predictive performance.

## Supplementary information

Supplemental Info

Supplemental Info
