# Supplementary material for: Machine learning metrology of cell confinement in melt electrowritten three-dimensional biomaterial substrates
Source: Microsyst Nanoeng. 2019 Mar 25;5:15. doi: 10.1038/s41378-019-0055-4 (PMC6431680; doi:10.1038/s41378-019-0055-4)
Supplement: Supplementary file 1 — Supplemental Info [file 41378_2019_55_MOESM1_ESM.pdf]

A) 3D Cell Reconstructions

Figure S1: 3D Cell reconstructions of controls (cells shown in Fig.4A)

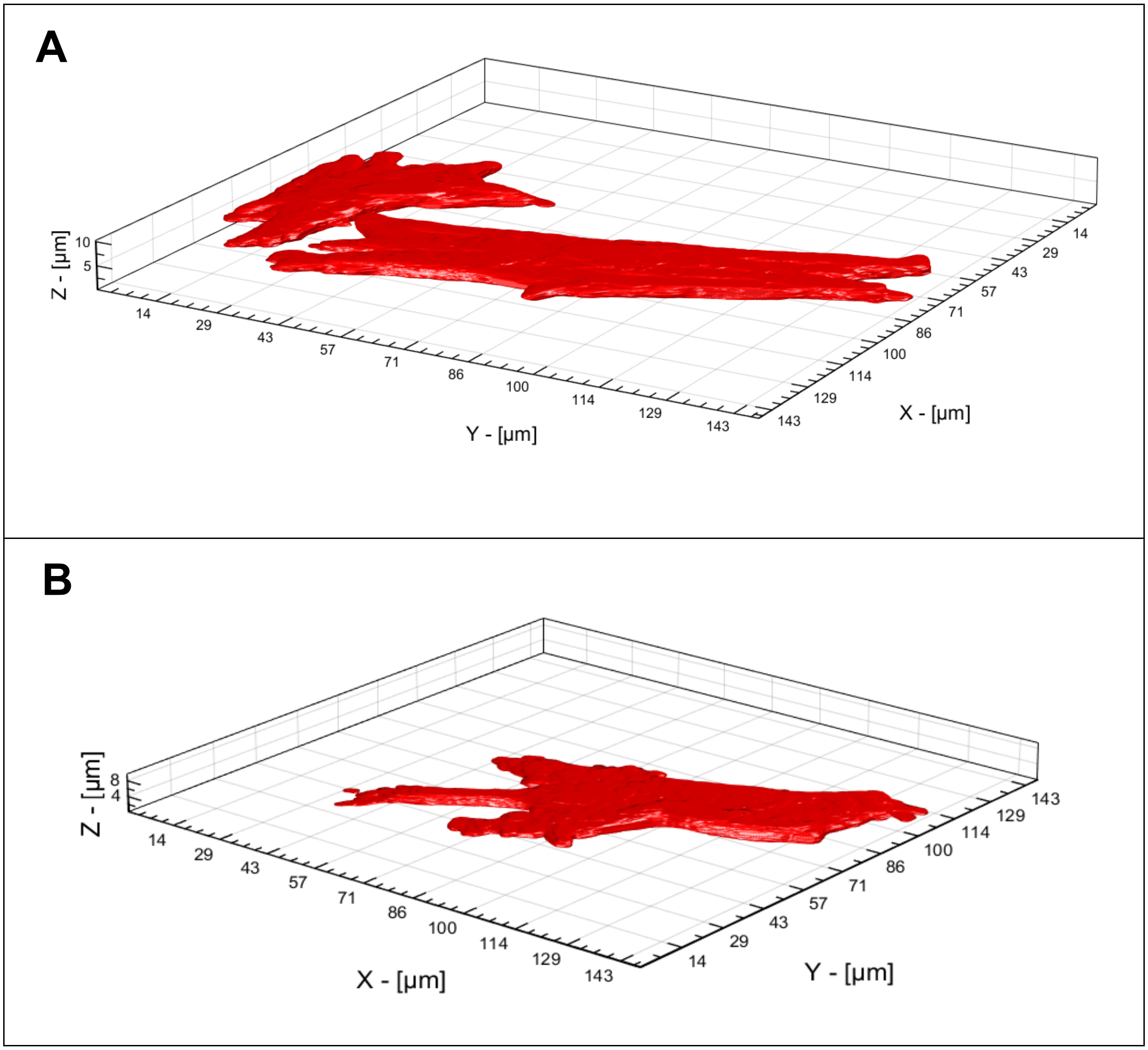

Figure S2: 3D Cell reconstructions of *SES-1 min* (cells shown in Fig. 4B)

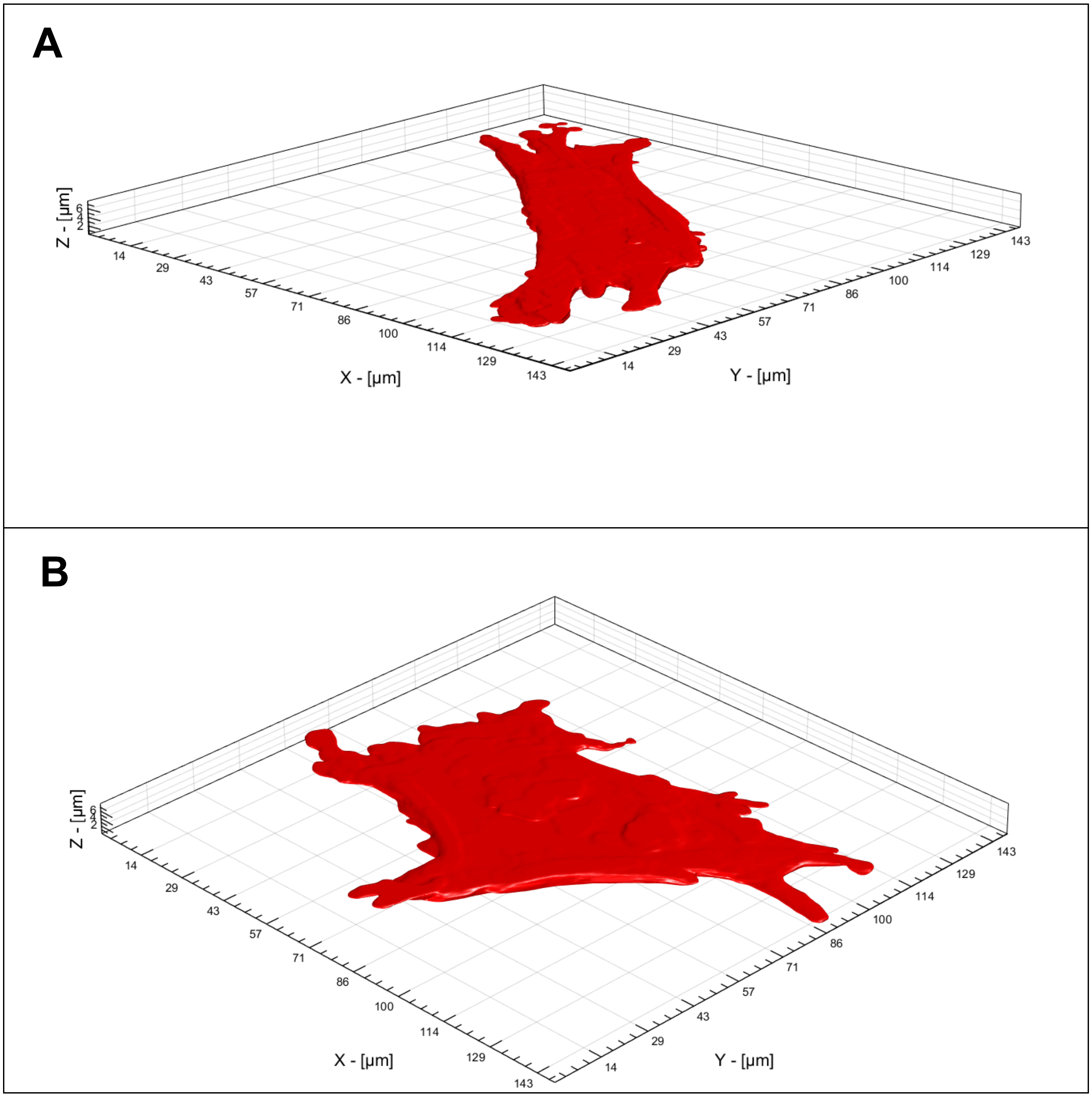

Figure S3: 3D Cell reconstructions of *SES – 3min* (cells shown in Fig. 4C)

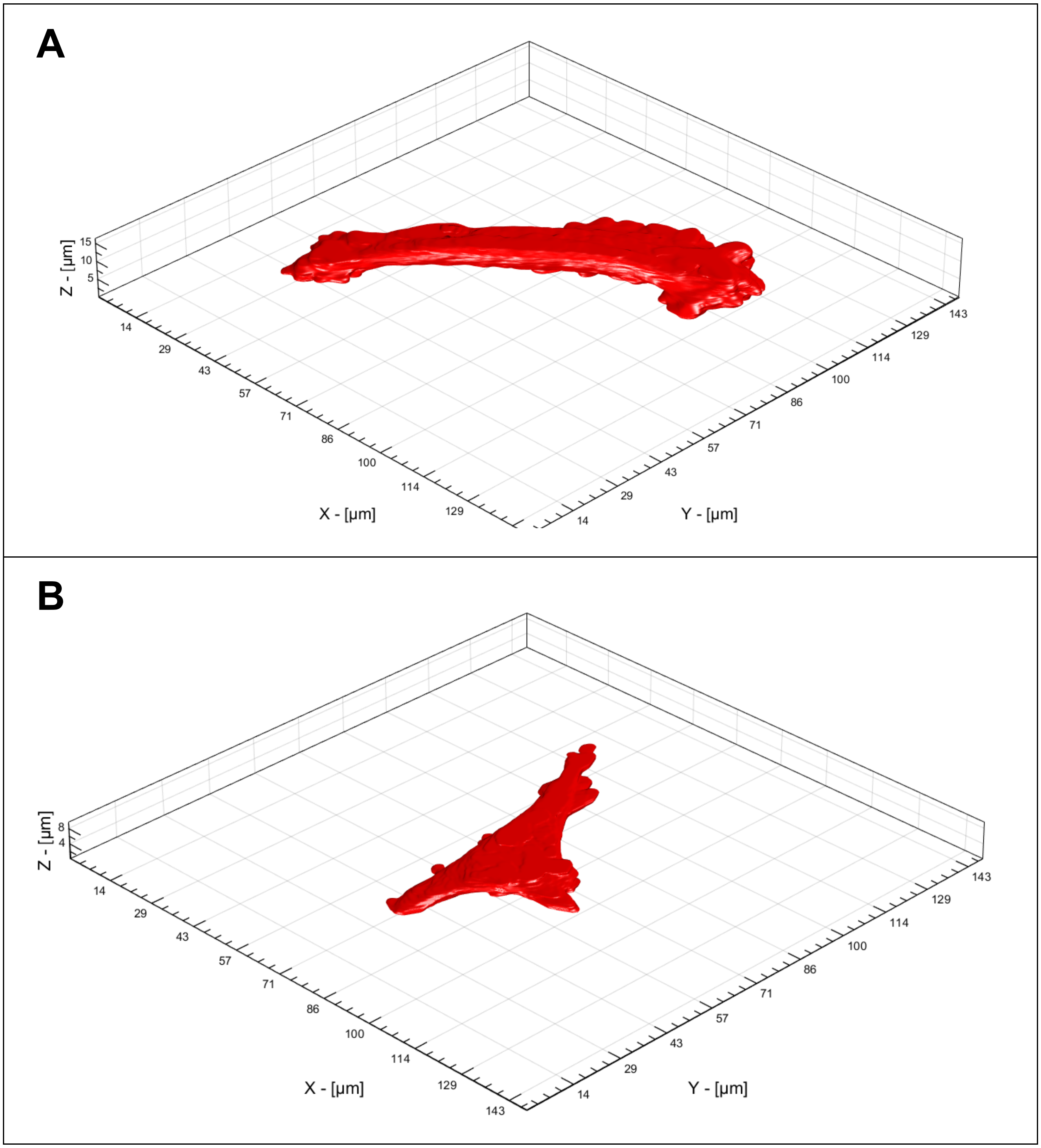

Figure S4: 3D Cell reconstructions of *MEW | 0-45°* (cells shown in Fig. 6)

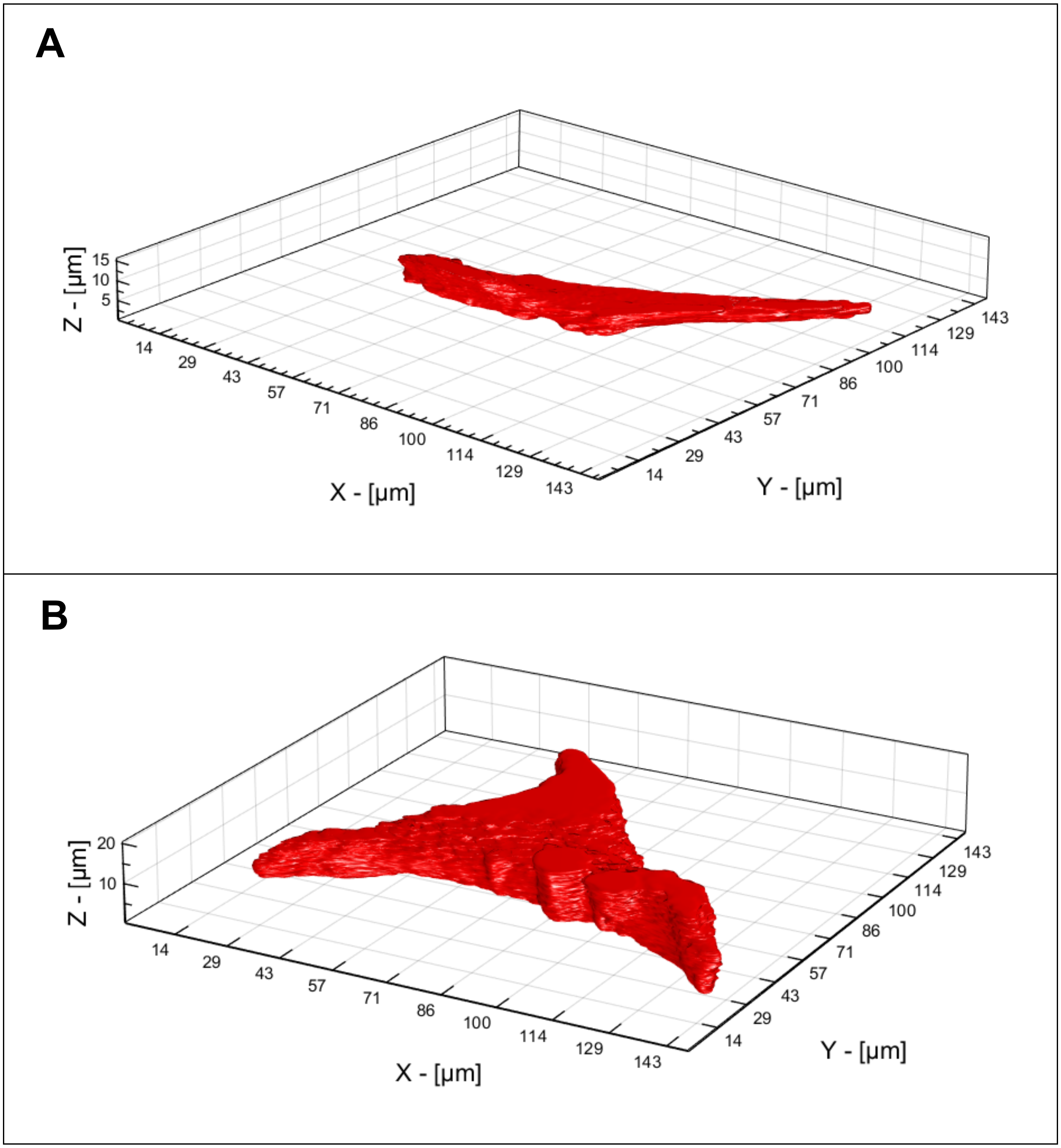

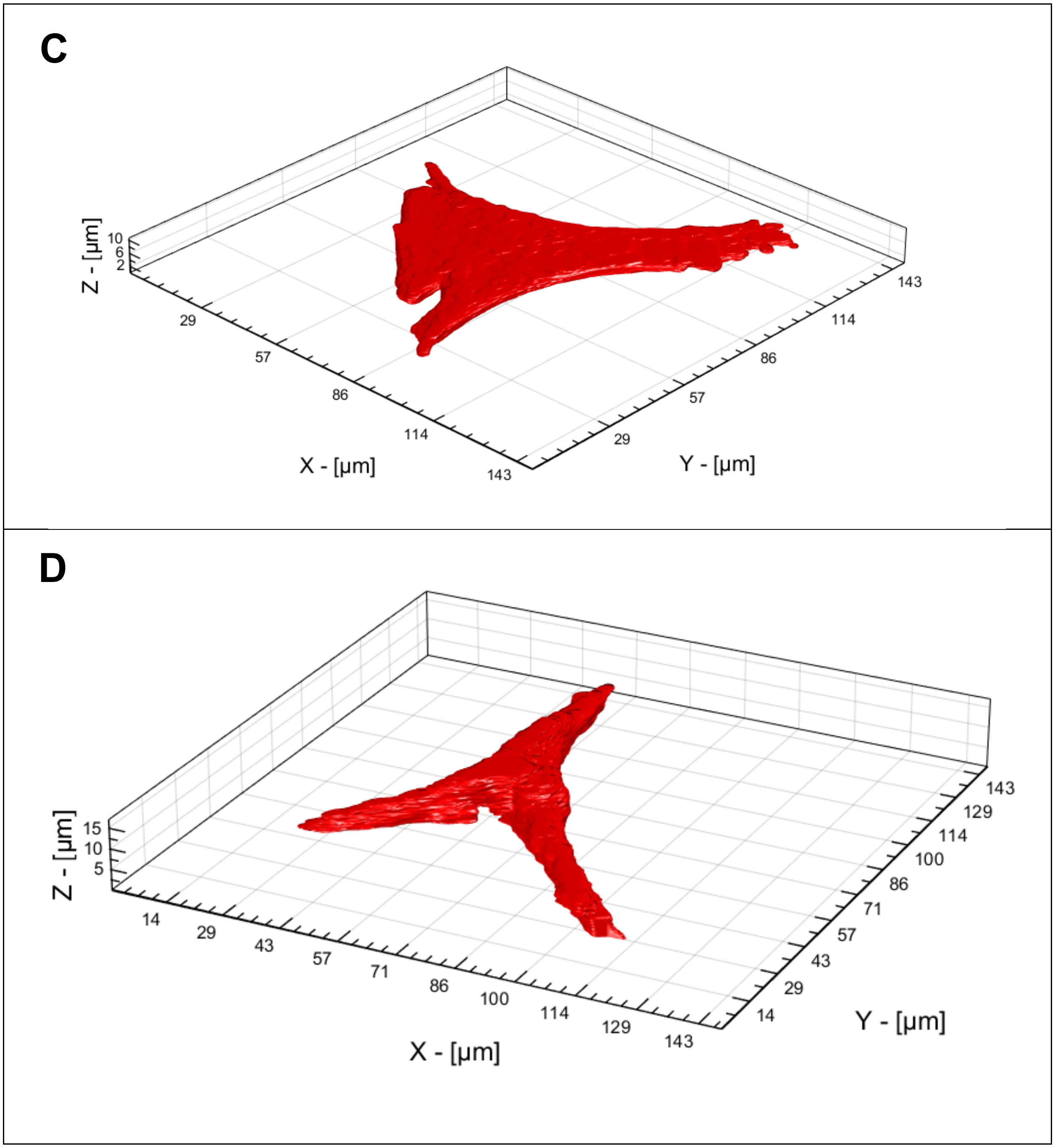
C
Z - [μm]
10
6
2
29
57
86
114
143
X - [μm]
29
57
86
114
143
Y - [μm]
D
Z - [μm]
15
10
5
14
29
43
57
71
86
100
114
129
143
X - [μm]
14
29
43
57
71
86
100
114
129
143
Y - [μm]

B) Image Processing Algorithmic Workflow for FA detection and sorting

The image processing algorithmic workflow for sorting FAs consists of two sequential main procedures (A and B) with each one having its own subsequent steps. Each step is described along with the its final outcome:

A) Detection of Cell Outline using the Red-Channel Maximum Projection.

1) Create the maximum projection Using the raw z-stack confocal images.	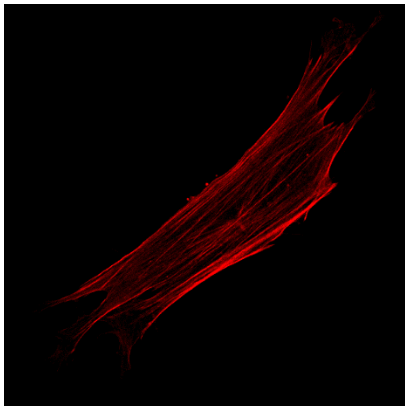<br>**Figure S5**: Result after Step A-1.
2) Use the grayscale image and apply a Contrast-Limited Adaptive Histogram Equalization (CLAHE). CLAHE equalizes image brightness and contrast across the image by performing different histogram scaling in each tile of a grid of tiles whose size is defined by the user.<br><br><u>Settings</u>:<br>Method: Uniform<br>Tiles :21<br>Enhancement: 0.01	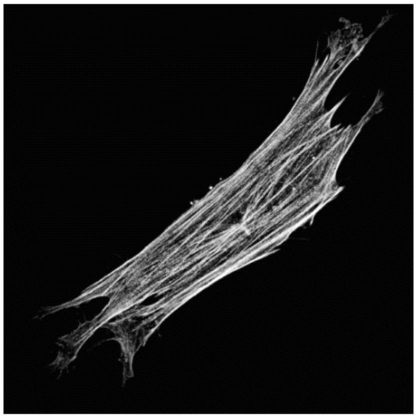<br>**Figure S6**: Result after Step A-2.

3) Apply “Adjust Contrast”. This step enhances image brightness and contrast by setting new white and black grayscale levels, and scaling the other levels accordingly.<br><br>Settings:<br>Mode: Auto<br>Low Level: 9<br>High Level: 83<br>Gamma: 1	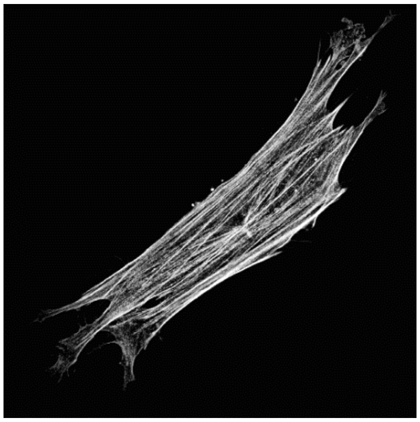<br>**Figure S7**: Result after Step A-3.

4) Basic Threshold. This step selects pixels based on whether they are below or above a certain pixel value. “Auto” determines this threshold value using Otsu’s method, which chooses the value which minimizes the average grayscale variance of the pixels which have been selected and not selected.<br>Settings:<br>Value: 4	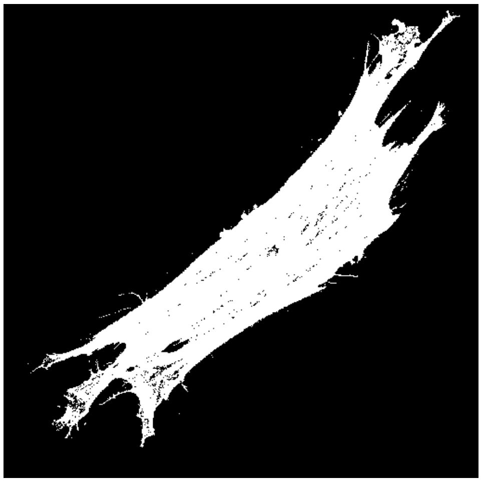<br>**Figure S8**: Result after Step A-4.

<table>
<tr><td>5) Apply “Smart Dilation”. This step selects pixels if they are surrounded by a number of selected pixels greater than or equal to the specified threshold number.

<u>Settings</u>:
Threshold: 8
Iterations: 10</td><td>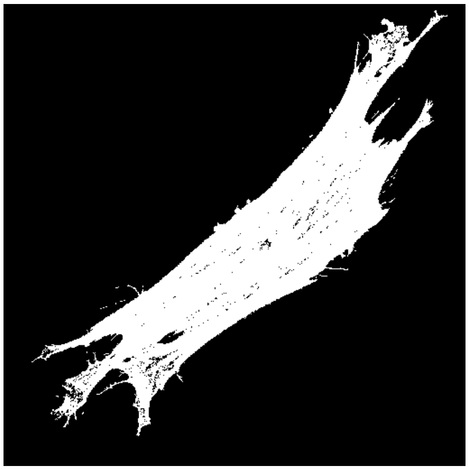</td></tr>
<tr><td></td><td>Figure S9: Result after Step A-5.</td></tr>
<tr><td>6) Apply “Smart Erosion”. This step Removes selected pixels if they are surrounded by a number of empty pixels greater than or equal to the specified threshold number.

<u>Settings</u>:
Threshold: 5
Iterations: 10</td><td>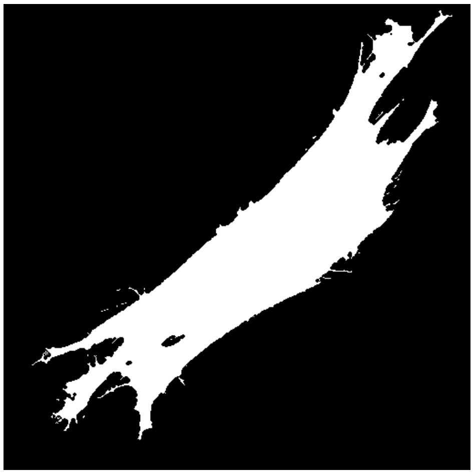</td></tr>
<tr><td></td><td>Figure S10: Result after Step A-6.</td></tr>
</table>

7) Reject Features. This step removes objects or fills holes based on their selected parameters being above or below a specified threshold. Parameter options include area, eccentricity, convex area/area ratio, and many more.<br><br>Settings:<br>Area = 20<br>Type: smaller or equal	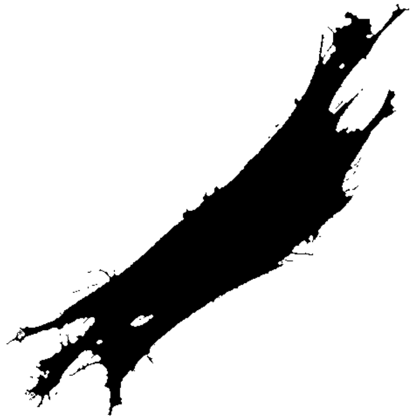<br>**Figure S11:** Result after Step A-7.
8) Reject Features<br><br>Settings:<br>Area = 50<br>Type: smaller or equal	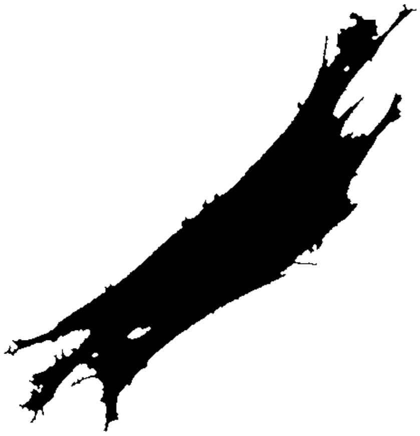<br>**Figure S12:** Result after Step A-8.

9) Reject Features

Settings:
Area = inf
Type: smaller or equal

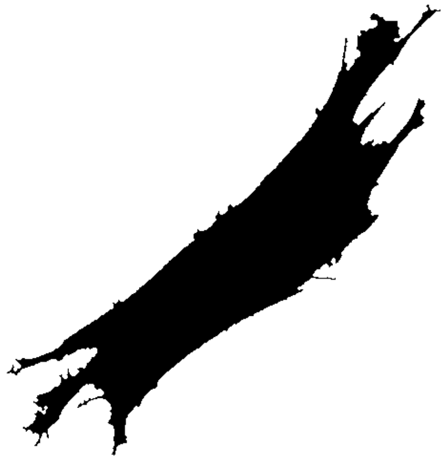

Figure S13: Result after Step A-9.

10) Apply "Smooth Features" This step smoothens feature boundaries using a specified window size. The threshold parameter controls the degree of feature erosion or dilation that also occurs.

i

ii

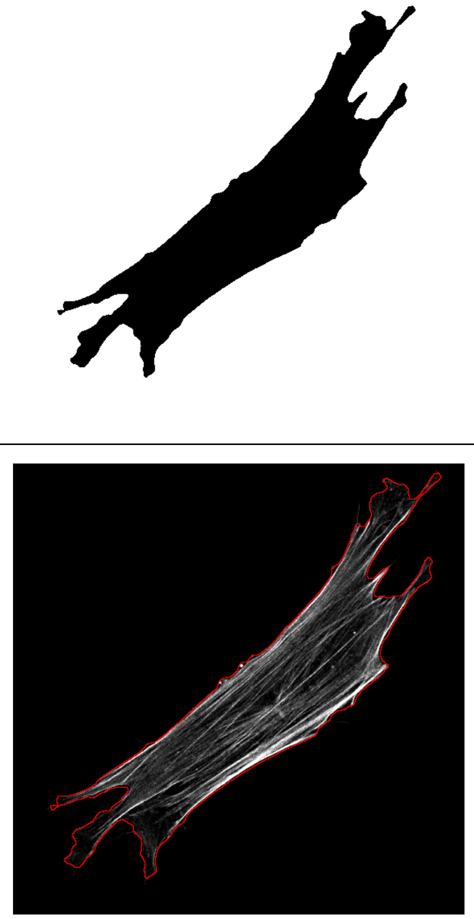

Figure S14: Result after Step A-10.

B) Detection of Adhesions within the Detected Cell Outline using the Green-Channel Maximum Projection.

1) Create the maximum projection Using the raw z-stack confocal images.	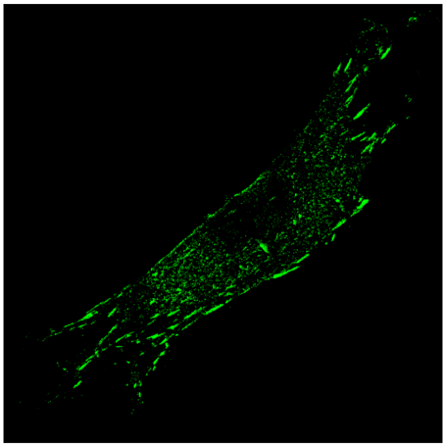<br>**Figure S15:** Result after Step B-1.

2) Adaptive Threshold using as a mask the previously detected cell outline (Figure shown in Step A-10)	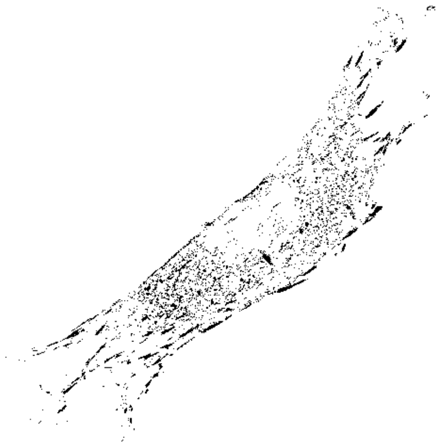<br>**Figure S16:** Result after Step B-2.

3) Smart Erosion	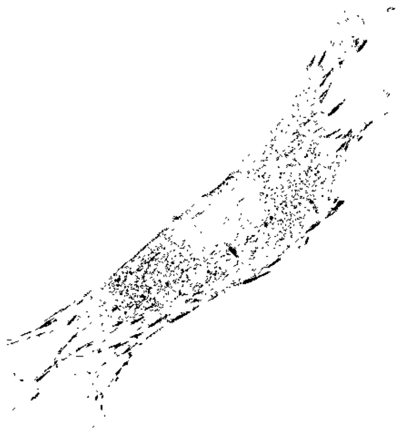
	**Figure S17:** Result after Step B-3.
4) Apply “Separate Features”. This step separates connected features using the watershed algorithm	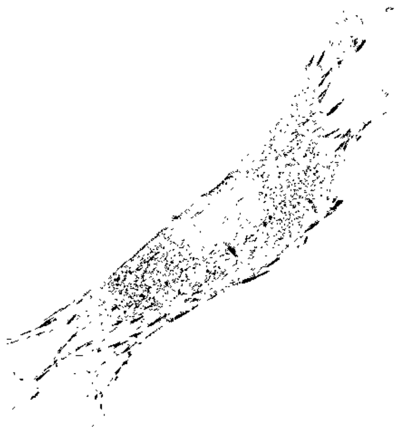
	**Figure S18:** Result after Step B-4.

<table>
<tr><td>5) Apply “Smart Dilation”
<u>Settings</u>:
Threshold = 7
Iterations = 10

And set this as the “Memory Image”</td><td>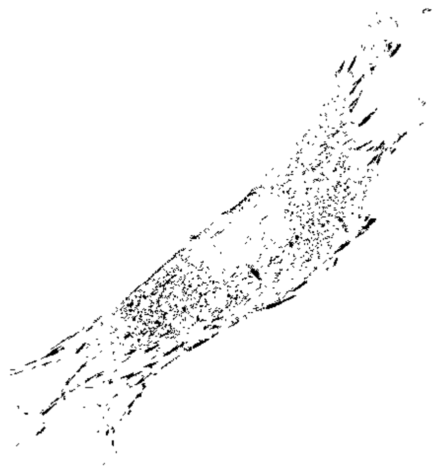
Figure S19: Result after Step B-5.</td></tr>
<tr><td>6) Reject Features all features with Area ≤ 0.2 μm²</td><td>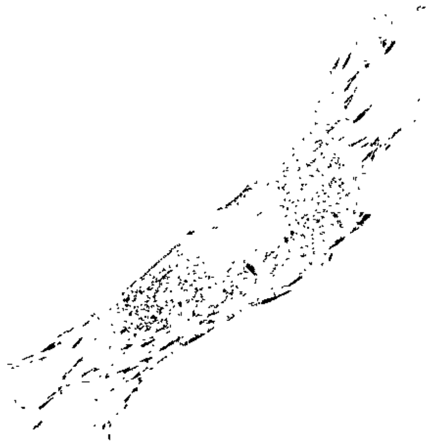
Figure S20: Result after Step B-6.</td></tr>
</table>

7) Rejected Features	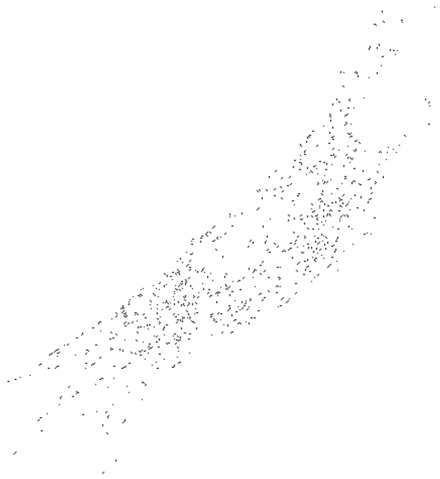
	**Figure S21:** Result after Step B-7.

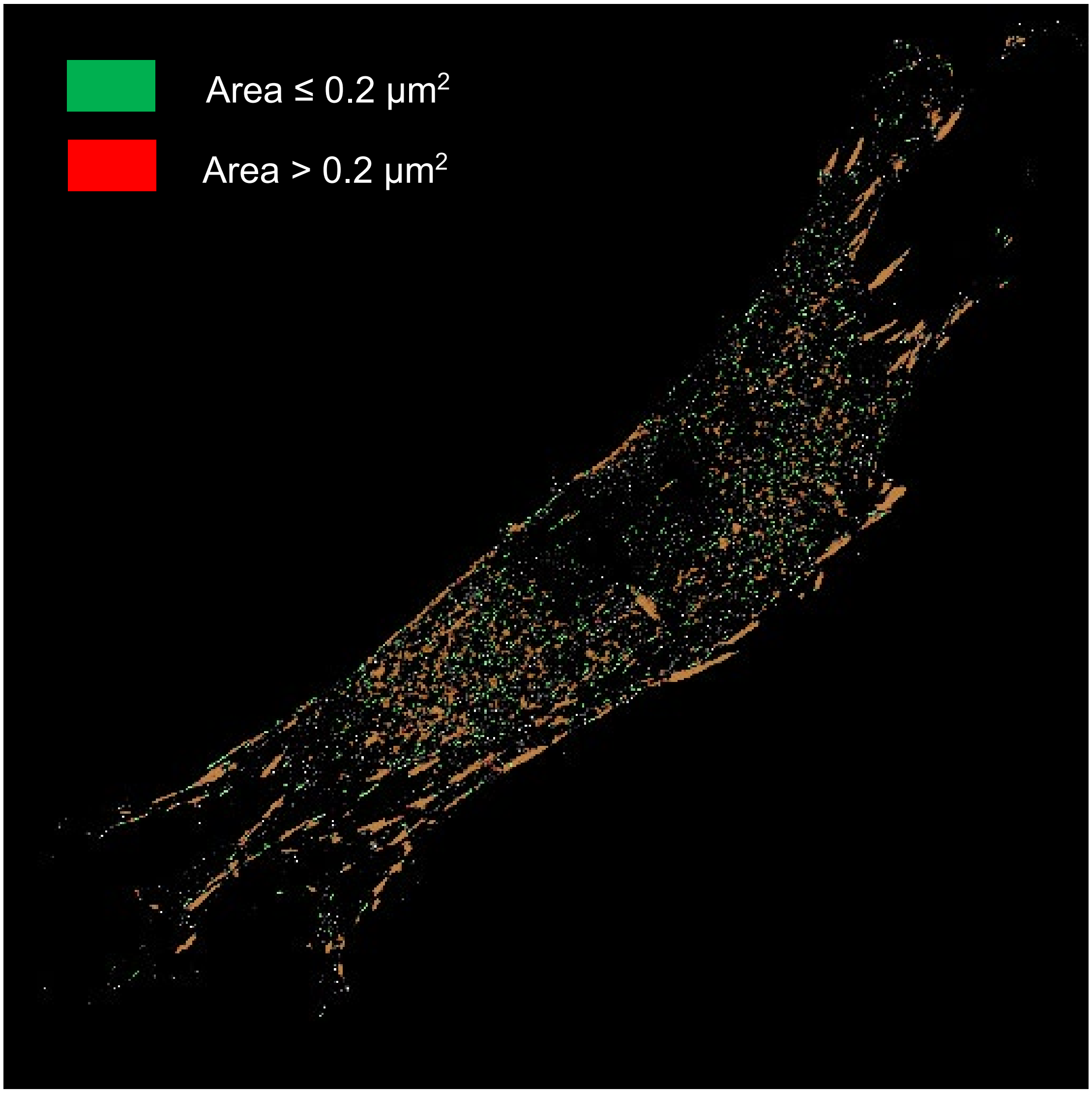

Figure S22: Final result of detected nascent (green) and mature (red) adhesions.
